# Astrocytic GPCR signaling in the anterior cingulate cortex modulates decision making in rats

**DOI:** 10.1093/oons/kvae010

**Published:** 2024-06-22

**Authors:** Mastura Akter, Zhongqi Fu, Xianlin Zheng, Zafar Iqbal, Na Zhang, Anwarul Karim, Ying Li

**Affiliations:** Department of Neuroscience, City University of Hong Kong, 83 Tat Chee Avenue, Kowloon Tong, Hong Kong, SAR, China; Department of Biomedical Sciences, City University of Hong Kong, 83 Tat Chee Avenue, Kowloon Tong, Hong Kong, SAR, China; Department of Neuroscience, City University of Hong Kong, 83 Tat Chee Avenue, Kowloon Tong, Hong Kong, SAR, China; Department of Biomedical Sciences, City University of Hong Kong, 83 Tat Chee Avenue, Kowloon Tong, Hong Kong, SAR, China; Department of Biomedical Sciences, City University of Hong Kong, 83 Tat Chee Avenue, Kowloon Tong, Hong Kong, SAR, China; Department of Neuroscience, City University of Hong Kong, 83 Tat Chee Avenue, Kowloon Tong, Hong Kong, SAR, China; Department of Biomedical Sciences, City University of Hong Kong, 83 Tat Chee Avenue, Kowloon Tong, Hong Kong, SAR, China; Centre for Regenerative Medicine and Health, Hong Kong Institute of Science & Innovation, Chinese Academy of Sciences, 15 Science Park West Avenue, Hong Kong Science Park, Pak Shek Kok, New Territories, Hong Kong, SAR, China; Hong Kong Institute of Science & Innovation, Chinese Academy of Sciences, 17W, Science Park West Avenue, Hong Kong Science Park, Pak Shek Kok, New Territories, Hong Kong, SAR, China; Department of Neuroscience, City University of Hong Kong, 83 Tat Chee Avenue, Kowloon Tong, Hong Kong, SAR, China; Department of Neuroscience, City University of Hong Kong, 83 Tat Chee Avenue, Kowloon Tong, Hong Kong, SAR, China; Department of Neuroscience, City University of Hong Kong, 83 Tat Chee Avenue, Kowloon Tong, Hong Kong, SAR, China; Department of Biomedical Sciences, City University of Hong Kong, 83 Tat Chee Avenue, Kowloon Tong, Hong Kong, SAR, China; Centre for Regenerative Medicine and Health, Hong Kong Institute of Science & Innovation, Chinese Academy of Sciences, 15 Science Park West Avenue, Hong Kong Science Park, Pak Shek Kok, New Territories, Hong Kong, SAR, China; Hong Kong Institute of Science & Innovation, Chinese Academy of Sciences, 17W, Science Park West Avenue, Hong Kong Science Park, Pak Shek Kok, New Territories, Hong Kong, SAR, China; Centre for Biosystems, Neuroscience, and Nanotechnology, City University of Hong Kong, 83 Tat Chee Avenue, Kowloon Tong, Hong Kong, SAR, China

**Keywords:** astrocyte, lactate, GPCR, decision making, anterior cingulate cortex

## Abstract

Decision making is a process of selecting a course of action by assessing the worth or value of the potential consequences. Rat Gambling Task (RGT) is a well-established behavioral paradigm that allows for assessment of the decision-making performance of rats. Astrocytes are emerging as key players in modulating cognitive functions. Using repeated RGTs with short intersession time intervals (48 h), the current study demonstrates that G_i_ pathway activation of astrocytes in the anterior cingulate cortex (ACC) leads to impaired decision-making in consistently good decision-making rats. On the other hand, ACC astrocytic G_q_ pathway activation improves decision-making in a subset of rats who are not consistently good decision-makers. Furthermore, we show that astrocytic G_q_ activation is associated with an increase in the L-lactate level in the extracellular fluid of the ACC. Together, these results expand our knowledge of the role of astrocytic GPCR signaling in modulating cognitive functions.

## INTRODUCTION

Decision making, a process by which humans and animals select a course of action by assessing the worth or value of their potential consequences [1], plays a vital role in our daily lives and is essential for adapting to our surroundings and maintaining independence [2]. Among the various higher-order cognitive functions that can be modeled in animals, decision making holds particular significance due to its culmination from the integration of multiple executive functions required for controlling and executing complex tasks [3]. Decision making requires the integration of sensory, affective, and cognitive processes [3]. In the field of psychiatry, bad decision-making capability is considered as mental illness [4]. Considering the fundamental role of decision making in daily life, a comprehensive neurobiological understanding of decision making is warranted.

IOWA Gambling Task (IGT) is an experimental neuropsychological task that assesses real-time decision making in humans based on the consequences of rewards and punishments [5]. Similarly, the Rat Gambling Task (RGT) is an analogous task for evaluating decision making in rats and allows for a rapid assessment of their performance [6]. It enables the differentiation between rats with good and poor decision-making abilities [6]. In RGT experimental design, the best decision is to control preference for a larger immediate gratification to maximize the long-term advantages. It is well established that the anterior cingulate cortex (ACC), a part of prefrontal cortex region, plays a crucial role in decision making [7–9].

Astrocytes, the predominant glial cells in the central nervous system, are crucial for maintaining the proper health and function of the nervous system. They provide metabolic and trophic support to neurons [10]. They express numerous receptors and transporters, and release gliotransmitters, playing a crucial role in sensing and modulating neuronal activity [11–14]. Activation of GPCRs by external stimuli like neurotransmitters or hormones triggers downstream signaling cascade including phospholipase C, adenylate cyclase, inositol 1,4,5-trisphosphate (IP3) [13]. Optogenetics and chemogenetics techniques are the most frequently used techniques to modulate the activity of neuronal and glial cells [15]. Designer Receptors Exclusively Activated by Designer Drugs (DREADDs) are a genetically modified chemogenetic technique to manipulate GPCRs of the CNS cells including astrocytes [16]. Previous study demonstrated that activation of astrocytic G_i_ pathway in the CA1 area of hippocampus (HPC) during learning leads to a particular difficulty in recalling remote memory of mice. This impairment of remote memory retrieval is accompanied by a decreased neuronal activity observed in the ACC during the retrieval phase [17]. Recently, we found that astrocytic G_i_ pathway activation in the ACC impairs schema memory in rats [18]. Other studies demonstrated that activation of astrocytic G_q_ pathway in anterior cortex of mice enhanced long term memory [19] and inhibition of astrocytic G_q_ pathway with iβARK impaired spatial memory in mice [20]. Here, we hypothesized that astrocytic G_i_ or G_q_ pathway in ACC might modulate the decision-making ability of rats.

We previously demonstrated that rats with chronic visceral hypersensitivity develop ACC astrogliosis and decision-making deficits. Additionally, we found that optogenetic activation of ACC astrocytes rescues the decision-making deficits by inducing L-lactate release in VH rats, underscoring the beneficial effect of L-lactate in decision making [21]. L-lactate, mainly produced by astrocytes through glycolysis and glycogenolysis, is recognized as a gliotransmitter that supports different cognitive functions [21–24]. Recently, we showed that astrocytic G_i_ activation in the ACC decreases the L-lactate level in the extracellular fluid (ECF) [18]. However, the effect of ACC astrocytic G_q_ activation in L-lactate level is unknown. G_q_ activation in astrocytes results in a consistent increase in spontaneous astrocytic Ca^2+^ events [25]. Ca^2+^ signals are key triggers for aerobic glycolysis in astrocytes resulting in increased L-lactate production and this process can be augmented by cAMP [26]. In this study, we demonstrate that ACC astrocytic G_q_ pathway activation increases L-lactate levels in the ACC ECF. Furthermore, we reveal that astrocytic G_i_ pathway activation leads to impaired decision-making in consistently good (i.e. advantageous) decision-making rats, while G_q_ pathway activation enhances decision-making in a subgroup of disadvantageous decision-making rats.

## RESULTS

### Expression of hM4Di or hM3Dq on ACC astrocytes

**Figure 1:**
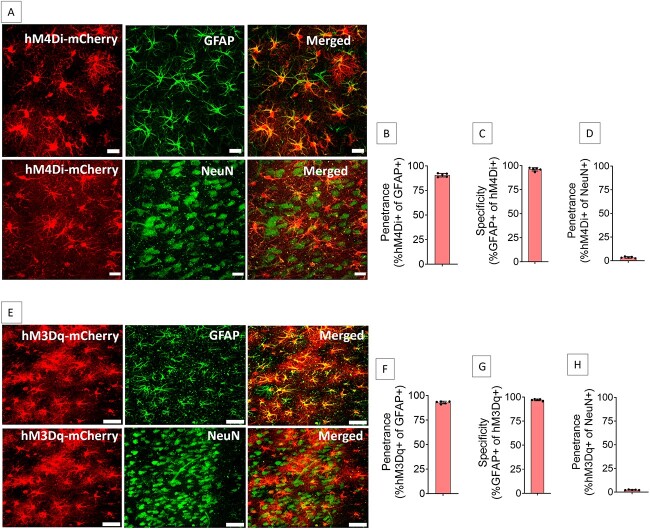
Expression of hM4Di or hM3Dq in the ACC astrocytes. (**A**–**D**) Injection of AAV8-GFAP-hM4Di-mCherry into ACC resulted in expression of hM4Di (A) in 90.6 ± 1.8% of GFAP-positive cells (B) with 96.1 ± 1.8% specificity (C), whereas 3.5 ± 0.8% of NeuN-positive cells expressed hM4Di (D). n = 5 rats. Scale bars: 20 μm. (**E–H**) Injection of AAV8-GFAP-hM3Dq-mCherry into ACC resulted in expression of hM3Dq (E) in 92.9 ± 1.3% of GFAP-positive cells (F) with 97.1 ± 0.9% specificity (G), whereas 2.4 ± 0.3% of NeuN-positive cells expressed hM3Dq (H). n = 5 rats. Scale bars: 20 μm

ACC of both sides were injected with Adeno-Associated Virus serotype 8 (AAV8) vector encoding mCherry-tagged hM4Di under the control of Glial Fibrillary Acidic Protein (GFAP) promoter to drive hM4Di expression in ACC astrocytes (AAV8-GFAP-hM4Di-mCherry). The hM4Di is a modified human muscarinic receptor M4 that has been engineered to be insensitive to the endogenous ligand acetylcholine but can be activated by its selective ligand clozapine-N-oxide (CNO) [27]. Injection of AAV8-GFAP-hM4Di-mCherry into ACC resulted in expression of hM4Di in ACC astrocytes (Fig. 1A and Supplementary Fig. 1) with high penetrance (90.6 ± 1.8%, Fig. 1B) and specificity (96.1 ± 1.8%, Fig. 1C). Penetrance in NeuN positive cells was low (3.5 ± 0.8%, Fig. 1D).

Similarly, AAV8-GFAP-hM3Dq-mCherry was injected bilaterally into the ACC. It resulted in expression of hM3Dq in ACC astrocytes (Fig. 1E and Supplementary Fig. 1) with high penetrance (92.9 ± 1.3%, Fig. 1F) and specificity (97.1 ± 0.9%, Fig. 1G). Penetrance in NeuN positive cells was low (2.4 ± 0.3%, Fig. 1H). There was no significant difference in the number of GFAP^+^ cells in the ACC due to hM4Di or hM3Dq expression groups compared to control groups (Supplementary Fig. 2).

### G_q_ pathway activation in ACC astrocytes increases L-lactate levels in ACC

**Figure 2:**
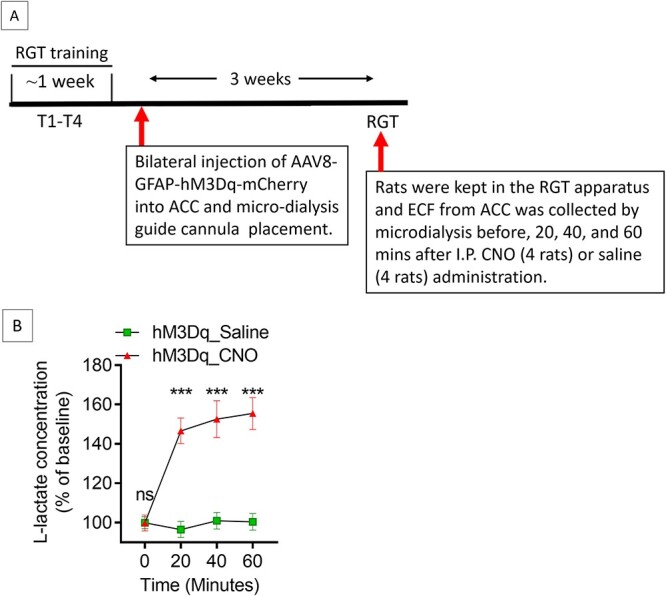
ACC astrocytic G_q_ activation increases L-lactate levels in ACC. (**A**) Experimental design to investigate the effect of G_q_ activation of ACC astrocytes on L-lactate levels. (**B**) Microdialysis measurement of L-lactate levels in the ECF of ACC before, 20 min, 40 min, and 60 min after intraperitoneal saline or CNO administration in hM3Dq expressed rats (n = 4 in each group). ^*^^*^^*^P < 0.001, ns = not significant, unpaired Student’s t-test

Recently, we showed that astrocytic G_i_ activation in the ACC of rats decreases the L-lactate levels in the ACC ECF [18]. To investigate the effect of ACC astrocytic G_q_ activation on L-lactate level, we prepared a cohort of eight rats (Fig. 2A). These rats received training (T1-T4) for RGT followed by bilateral injection of AAV8-GFAP-hM3Dq-mCherry into ACC. After three weeks, rats were placed in the RGT apparatus and ECF from ACC was collected by microdialysis before, 20, 40, and 60 min after I.P. CNO (3 mg/kg body weight, n = 4 rats) or saline (n = 4 rats) injection. As shown in Fig. 2B, we observed that L-lactate levels were significantly increased in the ECF due to G_q_ activation.

### Repeated RGTs with short intersession time intervals

**Figure 3:**
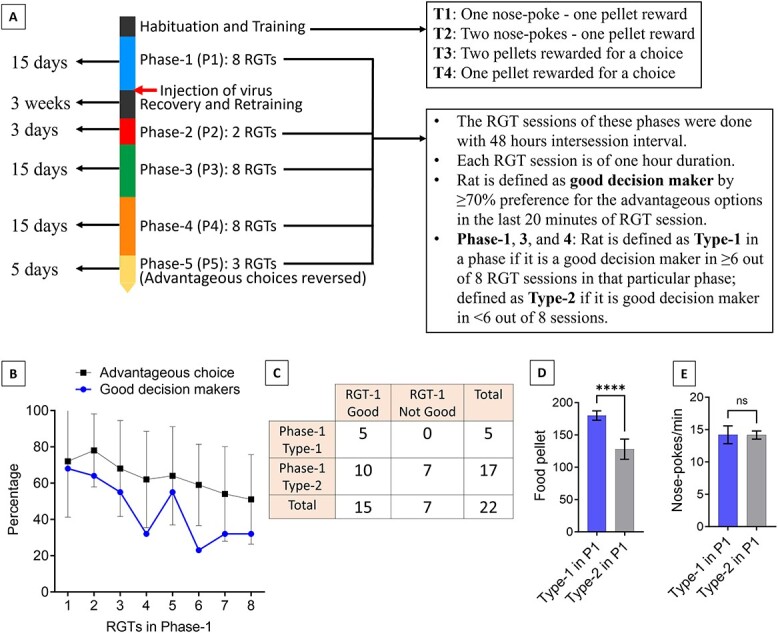
Repeated RGTs with short intersession time intervals. (**A**) Experimental design showing the timeline and phases of RGT sessions. (**B**) Mean (± SD) percentage of advantageous choices for all rats and proportion of good decision makers in eight RGTs of Phase-1. (**C**) Overall results of Phase-1. ‘RGT-1 good’ and ‘RGT-1 Not Good’: ≥70% and <70% preference for the advantageous choices in the first RGT, respectively. ‘Phase-1 Type-1’ and ‘Phase-1 Type-2’: Good decision-making behavior in ≥6 RGTs and <6 RGTs of Phase-1, respectively. (**D**) Mean food pellets obtained by both Type-1 and Type-2 rats during Phase-1. ^*^^*^^*^P < 0.001, unpaired Student’s t-test. (**E**) Mean nose pokes per minute of both Type-1 and Type-2 rats during Phase-1. ns = not significant, unpaired Student’s t-test

Previous study showed that the distribution of good and poor decision-making rats remains consistent if the RGT sessions are performed with long intersession time intervals (1.5 to 3 months) [6]. However, it is unknown whether this distribution remains consistent if the RGT sessions are performed with short intersession time intervals. As we wanted to investigate the role of ACC astrocytic G_i_ and G_q_ pathways in decision making longitudinally involving multiple RGT sessions, we first wanted to investigate how rats manifest their decision-making behavior if RGTs were done with short intersession time intervals. To this end, we performed eight RGTs with 48 h interval between successive sessions (Phase-1, Fig. 3A–E). Sixty-eight percent (15/22) of the rats were good decision makers (defined by ≥70% preference for the advantageous choices) in the first RGT (Fig. 3B and C). However, in contrast to the RGTs performed with long intersession time intervals where the proportion of good decision makers remained fairly consistent [6], we observed a gradual decrease in the preference of the advantageous choices throughout the eight RGTs from the whole cohort data along with a gradual decrease in the percentage of good decision makers over the eight RGTs. Although this discrepancy remains unexplained at this moment, this observation led us to classify the rats based on their consistency in the good decision-making behavioral manifestation in the eight RGTs for the next steps of our experiment. We defined rats to be consistently good decision makers if they were good decision maker in six or more out of the eight RGTs. Hereafter, they will be referred as Type-1 rats. Other rats (i.e. good decision-making in less than six out of eight RGTs) will be referred as Type-2 rats. With this criterion, 22.7% (5/22) were classified as Type-1 and the remaining 77.3% (17/22) were classified as Type-2 rats in Phase-1 (Fig. 3C).

The mean food pellets rewarded per RGT was significantly higher in the Type-1 rats compared to the Type-2 (180 ± 7.3 and 127.9 ± 15.7 respectively, unpaired Student’s t test, t = 7.11, df = 20, *P* < 0.001, Fig. 3D). The general activity as measured by the nose-pokes/min was not different between the Type-1 and the Type-2 rats (14.3 ± 1.3 and 14.2 ± 0.8 respectively, unpaired Student’s t test, t = 0.18, df = 49, *p* = 0.855, Fig. 3E).

### G_i_ pathway activation in ACC astrocytes can impair decision making

**Figure 4:**
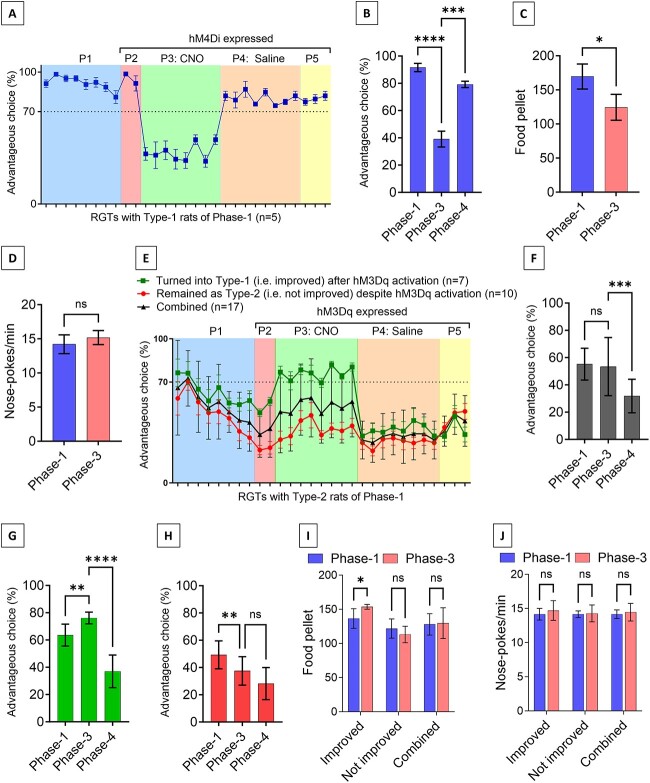
Astrocytic G_i_ pathway activation in ACC impairs decision making in RGTs whereas G_q_ pathway activation can improve decision making in a subgroup of rats. (**A–D**) Effect of ACC astrocytic G_i_ pathway activation in decision making. (A) Effect of ACC astrocytic G_i_ pathway activation in Type-1 rats identified in Phase-1 (n = 5). Astrocytic G_i_ activation with I.P. CNO administration dramatically impaired decision making in Phase-3 whereas the impairment was abolished in Phase-4 where I.P. saline was given. The percentage of advantageous choices remained unchanged despite reversal of advantageous choices in Phase-5. (B) Mean advantageous choices percentage per session for the Type-1 rats (n = 5) during Phase-1, Phase-3, and Phase-4. ^*^^*^^*^P < 0.001, ^*^^*^^*^^*^P < 0.0001, paired Student’s t-test, observed power for Phase-1 vs. 3 and Phase-3 vs 4 is >0.99 and >0.99, respectively. (C) Mean food pellets per session obtained by the Type-1 rats (n = 5) during Phase-1 and Phase-3. ^*^P < 0.05, paired Student’s t-test. (D) Mean nose pokes per minute of Type-1 rats (n = 5) during Phase-1 and Phase-3. ns = not significant, paired Student’s t-test. (**E–J**) Effect of ACC astrocytic G_q_ pathway activation in decision making. (E) Effect of ACC astrocytic G_q_ pathway activation in Type-2 rats identified in Phase-1 (n = 17). Seven rats (41.2%) turned into Type-1 rats (i.e. good decision making in ≥6 RGTs) in Phase-3 where astrocytic G_q_ pathway was activated with I.P. CNO administration. They turned back into Type-2 (i.e. good decision making in <6 RGTs) in Phase-4 where I.P. saline was given. The percentage of advantageous choices remained similarly poor during reversal of advantageous choices in Phase-5. Other rats (58.5%) remained as Type-2 in Phase-3. (F–H) Mean advantageous choices percentage per session during Phase-1, Phase-3, and Phase-4 for the whole cohort of Type-2 rats (n = 17), Type-2 rats turned into Type-1 in Phase-3 (n = 7), and Type-2 rats remained as Type-2 in Phase-3, respectively. ^*^^*^P < 0.01, ^*^^*^^*^P < 0.001, ^*^^*^^*^^*^P < 0.0001, ns = not significant, paired Student’s t-test, observed power for Phase-1 vs. 3 and Phase-3 vs 4 is 0.07 and >0.99 (F), 0.99 and >0.99 (G), 0.89 and 0.65 (H), respectively. (I) Mean food pellets per session obtained by the Type-2 rats during Phase-1 and Phase-3. ^*^P < 0.05, ns = not significant, paired Student’s t-test. (J) Mean nose pokes per minute of Type-2 rats during Phase-1 and Phase-3. ns = not significant, paired Student’s t-test

As we previously demonstrated that activation of ACC astrocytic G_i_ signaling decreases L-lactate level in the ACC [18], we hypothesized that astrocytic G_i_ activation in ACC might impair the decision-making ability of rats. To test this hypothesis, we used the Type-1 rats (n = 5) identified in Phase-1. After Phase-1, we injected AAV8-GFAP-hM4Di-mCherry bilaterally into the ACC to express the hM4Di receptor in the ACC astrocytes in these rats. After recovery and retraining for RGT, two RGTs (Phase-2) were done to check whether the surgery and expression of hM4Di affected their decision-making behavior. As shown in the Fig. 4A they still manifested good decision-making behavior. Then eight RGTs (Phase-3) were done with these rats. In Phase-3, they received I.P. CNO (3 mg/kg body weight, I.P.) 15 min before each RGT session to activate the G_i_ signaling pathway in the ACC astrocytes. Interestingly, their good (i.e. advantageous) decision-making ability was dramatically impaired, Phase-3, Fig. 4A. As a consequence, they collected significantly less food pellets in Phase-3 compared to Phase-1 (169.6 ± 18.3 and 124.4 ± 19 respectively, paired Student’s t test, t = 2.81, df = 4, *p* = 0.048) although their general activity was not impaired (Fig. 4C and D). We then withdrew the CNO and performed eight more RGTs (Phase-4) and observed that they reverted to being as consistently good decision makers (Type-1). To investigate whether their good decision-making ability was influenced by the position of advantageous choices, we reversed the position of advantageous choices and continued to do three more RGTs (Phase-5). They still demonstrated good decision-making ability indicating that they could identify the changes in advantageous choice positions and change their preference to the new advantageous choices (Fig. 4A).

### G_q_ pathway activation in ACC astrocytes can improve decision making

As we have demonstrated that activation of ACC astrocytic G_q_ signaling increases L-lactate level in ACC, we hypothesized that astrocytic G_q_ activation in ACC might improve decision-making. To investigate this, we injected AAV8-GFAP-hM3Dq-mCherry bilaterally into the ACC to express hM3Dq in the ACC astrocytes of Type-2 rats (n = 17) identified in Phase-1. In Phase-3, they all received CNO (3 mg/kg body weight, I.P.) before 15 min of each RGT session to activate the G_q_ signaling pathway in the ACC astrocytes (Fig. 4E). There was no significant improvement in the advantageous choices percentage in the whole cohort data of these Type-2 rats (Fig. 4E and F) as well as no differences in the food pellets awarded and nose pokes/min (Fig. 4I and J). However, when we sub-grouped the cohort based on whether the individual rats showed improved decision-making defined by becoming consistently good decision maker in Phase-3 (i.e. had good decision-making in ≥6 RGTs in the 8 sessions of Phase-3), we found that 41.2% (7/17) of the rats became consistently good decision makers in Phase-3 (Fig. 4E and G). As a result, the mean food pellets rewarded per RGT was significantly higher in Phase-3 compared to Phase-1 for these rats (153.6 ± 3.5 and 136.6 ± 14.4 respectively, paired Student’s t test, t = 3.33, df = 6, *P* = 0.016, Fig. 4I). Nose-pokes/min was not different between Phase-1 and Phase-3 for these rats indicating no impact on the general activity due to G_q_ activation (Fig. 4J). However, the remaining 58.8% (10/17) rats showed no improvement in decision-making and stayed as Type-2 rats in Phase-3 (Fig. 4E and H). We then withdrew the CNO and performed eight more RGTs (Phase-4). We observed that the subgroup-specific beneficial effect of astrocytic G_q_ activation on decision-making was abolished. This suggested that the beneficial effect of astrocytic G_q_ activation on decision-making is temporary and only apparent in a subgroup of disadvantageous decision makers.

One interesting observation was that the rats that transformed into consistently good decision makers due to G_q_ activation had slightly higher preference towards the advantageous choices in the Phase-1 compared to the other rats (mean advantageous choice 63.6 ± 8.1% and 49.2 ± 10% respectively, unpaired Student’s t test, t = 3.14, df = 15, *P* = 007). Although statistically not significant, these rats had slightly higher mean food pellets consumption per RGT in Phase-1 (136.6 ± 14.4 and 121.8 ± 14.1 respectively, unpaired Student’s t test, t = 2.11, df = 15, *P* = 0.052). The result might suggest that the rats having the least advantageous choice preferences in initial RGTs might be less sensitive to the beneficial effect of the G_q_ activation. Further studies with larger cohort size are needed to confirm this observation.

Finally, in Phase-5, we reversed the advantageous choices. The rats that showed no significant improvement in decision making due to G_q_ activation in Phase-3, now showed a higher advantageous choices percentage (Fig. 4E, Phase-5). This might suggest their previously developed preference towards the position of the prior disadvantageous choices. This this consistent with the previous reports where poor decision makers showed cognitive inflexibility by their persistence to choose the same location [28].

## DISCUSSION

Making a decision in complex and uncertain situations is a fundamental cognitive process that requires adaptation and integration of various executive functions [3]. Impaired decision making capabilities have been considered as a key symptom of many mental disorders [4]. The RGT enables researchers to study decision-making processes in rat animal models [6, 9, 21, 28]. A growing body of evidence support that astrocytes serve significant role in various cognitive functions [29, 30]. By exploring the role of astrocytes in cognitive functions, such as decision-making, we can gain novel neurobiological insights into these processes. This knowledge may potentially reveal new therapeutic approaches for treating disorders that impact cognitive functions.

Several studies have investigated the role of astrocytic G_i_ and G_q_ pathways in various cognitive functions in different brain areas. A study by Nam *et al*., showed that activation of astrocytic G_i_ in CA1 area of hippocampus improved contextual memory and synaptic plasticity [31]. In contrast, activating astrocytic G_i_ pathway in the CA1 during learning process impairs remote memory and decreases neuronal activity projecting from CA1 to ACC [17]. We recently found that astrocytic G_i_ pathway activation either in ACC or hippocampus impairs schema memory [18, 32]. Another study found that dorsal hippocampal astrocytic G_i_ pathway activation attenuates stress enhanced fear learning [33]. Moreover, striatal astrocytic G_i_ pathway activation corrected molecular abnormalities and rescued behavioral phenotype (anxiety, gait) in a Huntington’s disease mouse model [34]. However, the same group previously reported that astrocytic G_i_ pathway activation in striatum leads to hyperactivity and attention deficit in mice under normal physiological condition [35]. Another study found that cortical astrocytic G_q_ activation can longer sleep depth and duration in mice [36]. Together, these studies demonstrate that manipulation of astrocytic GPCR might have varying effects depending on the brain regions, cognitive functions, and the animal models investigated. In this study, we have shown that activation of astrocytic G_i_ signaling in the ACC impairs advantageous decision-making ability of rats, whereas G_q_ activation can enhance it in a subgroup of disadvantageous decision-making rats. However, further studies are required to elucidate the detailed molecular mechanisms responsible for these effects.

L-lactate in brain, predominantly originating from astrocytes and previously considered as metabolic waste product, is now recognized as a beneficial substance acting as a fuel and signaling molecule [22, 37]. Recent studies from our lab and others have highlighted the role of L-lactate in cognitive functions [18, 38–40] and the involvement of astrocytic pathways in its regulation [18, 21, 40]. We recently demonstrated that astrocytic G_i_ pathway activation in the ACC impairs paired associates learning and schema memory formation by decreasing L-lactate level in the ACC ECF which was associated with a decrease in the astrocytic cAMP upon G_i_ activation [18]. Furthermore, we previously reported impaired decision-making in visceral hypersensitive rats resulting from a decrease in ACC L-lactate levels [21, 41]. Therefore, we speculate that the impaired decision-making observed upon astrocytic G_i_ activation in our current study is mediated by a decrease in L-lactate level in the ACC. In this study, we also found that astrocytic G_q_ pathway activation increases L-lactate level in the ACC. Although the detailed mechanisms by which astrocytic GPCRs regulate L-lactate level are yet to be elucidated, it is well established that astrocytic G_q_ activation results in a consistent increase in spontaneous astrocytic Ca^2+^ events [25], whereas, astrocytic G_i_ activation induces a decrease in cAMP [18] and a transient rise followed by a decrease in intracellular Ca^2+^ [17]. Activation of hippocampal astrocytic cAMP enhanced synaptic plasticity and memory by increasing release of L-lactate from astrocytes [42]. Another study demonstrated that Ca^2+^ signals are key triggers for aerobic glycolysis in astrocytes resulting in increased L-lactate production and this process can be augmented by cAMP [26]. Based on this accumulated evidence, it is tempting to speculate that the decrease in cAMP upon astrocytic G_i_ activation and the increase in Ca^2+^ events upon astrocytic G_q_ activation are the likely mediators of the decrease and increase in L-lactate levels in the ACC ECF, respectively. Additionally, astrocytic G_q_ pathway activation was shown to increase ECF glutamate level in the nucleus accumbens core of rats which was dependent on astrocytic calcium signaling [43]. Astrocytes take up glutamate by EAAT2 (astrocytic glutamate transporter) to protect neurons from excitotoxicity. To uptake glutamate, astrocytes require high levels of energy which causes increased glycolysis and glycogenolysis resulting in L-lactate production [44, 45]. In line with this, a study demonstrated that the application of glutamate or electrical stimulation to cultures containing a mixture of neurons and astrocytes resulted in a notable increase of lactate levels in the surrounding media [46]. While direct *in vivo* investigations linking these findings are limited, these studies suggest that astrocytic G_q_ pathway may increase extracellular glutamate which may further stimulate astrocytic glycolysis and L-lactate production. Further studies are needed to investigate these potential hypotheses and better understand the molecular mechanisms that regulate the ECF L-lactate level upon different astrocytic GPCR signaling activation.

In a recent study, we showed that activating astrocytic G_q_ signaling in the ACC enhanced myelination and oligodendrogenesis in visceral hypersensitive rats [47]. Myelination plays a crucial role in different types of cognition including in decision-making behavior [47–49]. Previous study showed that the application of 1,4-dideoxy-1,4-imino-d-arabinitol, an inhibitor of glycogen phosphorylase, to block the *in vivo* production of L-lactate decreased remyelination in the corpus callosum in a mouse model with cuprizone-induced demyelination [50]. Moreover, it was demonstrated that, L-lactate rescues myelination in cultured slices of cerebral cortex of mice cultivated in low glucose conditions [51]. These studies suggest the importance of L-lactate in the process of myelination. Furthermore, astrocytes and oligodendrocytes contribute significantly to shuttle L-lactate into neuron for neuronal metabolic support [52]. Additionally, oligodendrocytes themselves utilize L-lactate as a source of energy [53, 54]. Thus, it remains an open question whether the enhanced decision-making observed upon astrocytic G_q_ activation in a subgroup of disadvantageous decision-making rats, might have been mediated by enhanced myelination in the ACC due to increased L-lactate level. It is also unclear why the other rats did not show improvement in the advantageous choice preference. One limitation of our current study is that we did not exclude the possibility of differential expression of hM3Dq in these two types of rats. Further studies are needed to better understand whether these results represent true biological variations among animals or due to difference in the expression level of the hM3Dq.

Increased plasma lactate due to exercise or intraperitoneal lactate injection can promote mitochondrial biogenesis in the hippocampus of mice [55]. Similarly, in a recent study we found that L-lactate administration into hippocampus can enhance mitochondrial biogenesis and antioxidant defense [56]. We also demonstrated that astrocytic G_i_ pathway activation in ACC reduces neuronal mitochondrial biogenesis whereas administration of L-lactate into ACC rescues the impairment [18]. Mitochondrial biogenesis is also known to enhance learning and memory in mice [57]. Therefore, future studies could investigate whether mitochondrial biogenesis plays role in decision-making.

One important consideration while interpreting results obtained with DREADD and CNO system is the potential off-target effects of CNO. Gomez *et al*. investigated the mechanism of action of CNO in DREADD-expressing animals [58]. They found that upon systemic injection of CNO it is converted into clozapine that crosses the blood–brain barrier and binds to DREADDs expressed in the central nervous system. The study showed that I.P. injection of 0.1 mg/kg clozapine (which is equivalent to 10 mg/kg CNO) decreases locomotor activity only in the hM4Di-expressing (in basal forebrain) rats but not in control rats without hM4Di expression. In our study, we used 3 mg/kg I.P. CNO (expected to be equivalent to 0.03 mg/kg clozapine) which is below the level at which the study did not find DREADD-independent effect. Furthermore, although in the current study we did not use a control group to investigate if CNO alone has off-target effects, our previous study using the same dose showed that it does not affect the paired-associate learning and schema memory formation as well as the general activity in rats [18]. This is also consistent with other reports which failed to find significant effects of up to 10 mg/kg CNO on various motivated behaviors in non-DREADD-expressing animals, at least within a 30–150 min timeframe after I.P. injection [59, 60]. Therefore, the effects observed in our current study is unlikely to be due to non-specific or off-target effects of CNO as we have used a dose of 3 mg/kg and the RGT sessions were started after 15 min and lasted for only 60 min.

While our current study design allowed us to observe the effect of G_i_ or G_q_ activation/inactivation through three key phases (Phase-1, Phase-3, and Phase-4) of RGTs, it would be further informative to investigate how different GPCR manipulations affect the decision-making in longer terms when the advantageous choices are reversed (currently only 3 RGTs were done in Phase-5 where the advantageous positions were reversed). Moreover, we did not investigate how different GPCR manipulations during the RGT training (T1-T4) affect subsequent decision-making in the RGT sessions. Further studies are needed to address these limitations.

In summary, the present study illustrates that ACC astrocytic G_i_ pathway activation impairs advantageous decision-making whereas G_q_ pathway activation increases L-lactate level in the ECF of ACC and may improve decision making in a subgroup of disadvantageous decision-making rats. These results expand our knowledge of the role of astrocytic GPCR signaling in modulating cognitive functions.

## METHODS

### Animal use and care

Adult male Sprague–Dawley rats weighting about 250–300 g were used in this study. All rats were housed in a standard laboratory facility (25°C, 50% humidity, 12-h light/dark cycle with light on at 7:00 AM). All animals were supplied by the Laboratory Animal Services Centre, Chinese University of Hong Kong. All experimental procedures using animals were conducted according to the guidelines developed by the Committee on Use and Care of Animals, Department of Health, Govt. Hong Kong SAR. The License numbers to conduct experiments are: (22–2) in DH/HT&A/8/2/5 Pt.8 and (22–3) in DH/HT&A/8/2/5 Pt.8. The approval for ‘Ethical Review of Research Experiments involving Animal Subjects’ were taken by Animal Research Ethics Sub-Committee, City University of Hong Kong (References: A-0513 and A-0417).

### Rat gambling task protocol

The decision-making capability of rats can be detected by the rat gambling task (RGT) [6]. We prepared rats for RGT as was described in our previous publications [9, 21, 61]. After food restriction for three days followed by one day of fasting, training sessions for RGT was done. Daily food restriction was continued throughout the training. For training-1 (T1), rats were trained to associate a single nose-poke with one food pellet delivery with a criterion of having obtained 100 pellets within a 30-min session. In T2, rats were trained to associate two consecutive nose-pokes with one food pellet reward with the same criterion. After completing T1 and T2, two 5 min sessions (T3 and T4) were conducted to habituate rats for the variation in the number of rewarded food pellets. In T3, two pellets were rewarded after a choice was made, whereas in T4, one pellet was rewarded. After completion of training, RGT sessions were performed from the following day with 48-h intersession time interval.

RGT sessions are 60-min testing sessions where rats were free to make choices among four holes (A–D). Choices A and B were disadvantageous choices for which two pellets were delivered each time as immediate reward but had separately 1/2 probability to trigger a long penalty time-out (222 s) or 1/4 probability for a very long penalty time-out (444 s) during which no pellet can be obtained. Choices C and D were advantageous choices for which one pellet was delivered as immediate reward but had smaller penalty time-out (1/4 chance for 12 s time-out, or 1/2 chance for 6 s time-out). Note that the selected hole remained illuminated during penalty period to facilitate the association between the selection and its outcome and no food pellet could be obtained during the penalty period. A rat was defined as good decision maker by ≥70% preference for the advantageous options in the last 20 min of RGT sessions. The equation is as follows:


\begin{align*} &\mathrm{Percentage}\ \mathrm{of}\ \mathrm{advantageous}\ \mathrm{choices}=\\&\frac{Choices\ in\ \left(C+D\right)}{Choices\ in\ \left(A+B+C+D\right)}\times 100\% \end{align*}

To identify consistently good decision-making rats, we performed eight consecutive RGTs with 48-h intersession time interval (Phase-1). Moderate food restriction was applied on the day before each RGT session. The rats that showed good decision-making behavior in at least six out of eight RGTs were considered as consistently good decision makers (referred as Type-1 rats). Other rats (i.e. good decision making in less than six out of eight RGTs) were referred as Type-2 rats.

Next, we investigated the effect ACC astrocytic G_i_ or G_q_ pathway activation on decision making (Fig. 3A). For studying the effect of G_i_ activation, AAV8-GFAP-hM4Di-mCherry was injected bilaterally into the ACC (procedure is described later) in a subset of Type-1 rats identified in Phase-1. For studying the effect of G_q_ activation, AAV8-GFAP-hM3Dq-mCherry was injected bilaterally into the ACC in a subset of Type-2 rats identified in Phase-1. Three weeks later, two RGT sessions (Phase-2) were done for these rats. Then Phase-3 was started where eight RGTs were done with 48-h intersession time intervals. In this phase, rats received CNO (3 mg/kg body weight, I.P.) 15 min before each RGT. Next, eight more RGTs were performed (Phase-4) without CNO administration. Finally, in Phase-5, three RGTs were done where the advantageous choices were reversed (i.e. advantageous choices: A and B, disadvantageous choices: C and D). The equation for calculating the percentage of advantageous choices in the RGTs of this phase was as follows:


\begin{align*} &\mathrm{Percentage}\ \mathrm{of}\ \mathrm{advantageous}\ \mathrm{choices}=\\&\frac{Choices\ in\ \left(A+B\right)}{Choices\ in\ \left(A+B+C+D\right)}\times 100\% \end{align*}

### Stereotactic surgical procedures, viral vector injection, and CNO administration

To express hM4Di or hM3Dq in the ACC astrocytes, AAV8-GFAP-hM4Di-mCherry or AAV8-GFAP-hM3Dq-mCherry was used (original viral titer 3 × 10^12^ vg/ml diluted in 1:10 in PBS, TaiTool and Vigene Bioscience Corp. Ltd respectively). Rats were anesthetized with 50 mg/kg I.P. sodium pentobarbital (Dorminal 20%, Alfasan International BV, Woerden, Holland, Cat #: 013003) administration and placed in a stereotaxic frame. After exposing the skull, bilateral craniotomy was done (0.5–0.8 mm holes, 2.2–3.8 mm anterior to bregma, 0.5–1.0 mm lateral from midline). A 10 μl micro-syringe (Hamilton, NV, USA) with a 33-gauge metal needle was used to perform the microinjections. We injected 400 nl of viral vector bilaterally into the ACC regions (2–3 mm ventral from the surface of the skull at the craniotomy site) with injection flow rate of 0.1 μl/min (controlled by microinjection pump, World Precision Instruments, USA). The needle was left in place for an additional 5 min after the injection was completed. Then it was slowly withdrawn. After withdrawing the needle, the scalp was sutured, and immediate postoperative care was provided with local anesthetic (xylocaine, 2%) applied to the incision site for analgesia and allowing the rats to recover from anesthesia under a heat pad. The rats were returned to their home-cage after awaking. All rats were allowed three weeks of rest to ensure high level of hM4Di or hM3Dq expression.

Clozapine-N-oxide (CNO) dihydrochloride (Hello Bio, Avonmouth, UK, Cat #: HB6149), a synthetic ligand to activate hM4Di or hM3Dq, was dissolved in 0.9% NaCl and was injected intraperitoneally (I.P.) at a dose of 3 mg/kg body weight. This dose did not produce any seizure in rats.

### Measurement of L-lactate levels

To investigate the effect of ACC astrocytic G_q_ pathway activation on L-lactate level in the ACC, 8 rats underwent four RGT training sessions as shown in Fig. 2A. Then bilateral AAV8-GFAP-hM3Dq-mCherry injection into the ACC was done as described before in all rats. In addition, a micro-dialysis guide cannula (CMA Inc.) was inserted into the right sided ACC (2.5 mm ventral from the surface of the skull at the craniotomy site) in the rats that was used for microdialysis later (eight rats). After three weeks, rats were given I.P. CNO (3 mg/kg body weight) (n = 4 rats) or saline (n = 4 rats) and placed in the RGT apparatus. Extracellular fluid (ECF) from ACC was collected before, 20, 40, and 60 min after CNO or saline administration.

The dialysates collected from ACC were kept at −80°C until further use. Lactate Fluorescence Assay kit (Abcam, USA, Cat #: ab65331) was used to determine the L-lactate concentration from the same ACC dialysate according to the manufacturer’s protocol.

### Immunohistochemistry and confocal microscopy

After completing experiments, rats were anesthetized by urethane (1.5 g/kg, I.P.) and perfused transcardially with ice-cold PBS for approximately 5 min and then perfused with 4% paraformaldehyde (PFA). The whole brain was taken out and postfixed in 4% PFA overnight at 4°C and cryoprotected in 30% sucrose dissolved in 1X PBS for an additional 3 days at 4°C. The brains were then stored in OCT medium at −80°C until further use. For IHC, each brain was sectioned at 40 μm using cryostat (Leica, USA) and processed as free-floating sections. Three to five sections were selected for staining per rat. Sections were incubated with blocking solution of Triton X-100 (0.3% [v/v]) and 10% normal goat serum (NGS) in 0.01 M PBS for 1 h at room temperature after a brief wash. Then sections were incubated with primary antibodies Anti-GFAP, Mouse Monoclonal, (Cat #: ab4648, Abcam), Anti-NeuN, Rabbit Polyclonal, (Cat #: AB978, Merck Millipore) in blocking solution for overnight at 4°C. In the following day, slices were washed 3 times (5 min each) and incubated with targeted Alexa flour secondary antibodies (1: 300) in DAPI (4′,6-diamidino-2-phenylindole) for 2 h at room temperature. Then the sections were mounted into microscopic slides (Epredia™ SuperFrost Plus™ Adhesion Microscopic Slides) and covered with coverslips (Eprdia Cover Slip) along with fluorescent mounting medium (DAKO). The imaging was done by inverted laser scanning confocal microscope (LSM 880; Carl Zeiss, Oberkochen, Germany). The confocal images for quantitative analysis were acquired under 20X or 40X oil-immersion objectives and analyzed with ImageJ.

### Data analysis

RGT data analysis was done in R Programming Environment (R version 4.0.2 in RStudio v1.3.959-1). Other data analyses were done with Prism v.7.0 (GraphPad Software, La Jolla, CA, USA) or MS Excel. Data are presented as mean ± SD as appropriate. Comparisons of continuous data were done with two-tail Student’s t test where appropriate. The observed power of key statistical tests was calculated with pwr.t.test function of pwr R package. Image analysis was done with ImageJ. Figures were generated with Prism v.7.0.

## ABBREVIATIONS

**AAV8**: Adeno-associated viral vectors serotype 8; **ACC**: Anterior cingulate cortex; **RGT**: Rat gambling task; **IGT**: Iowa gambling task; **VH**: Visceral hypersensitive; **ECF**: Extracellular fluid; **cAMP**: Cyclic adenosine monophosphate; **CNO**: Clozapine-N-oxide; **DREADD**: Designer receptors exclusively activated by designer drug; **ECF**: Extracellular fluid; **GFAP**: Glial fibrillary acidic protein; **GPCR**: G-protein-coupled receptor; **PBS**: Phosphate buffered saline; **PFA**: Paraformaldehyde.

## STUDY FUNDING

This work was funded by the General Research Fund (GRF) of the Research Grants Council of Hong Kong (11103721, 11102820, and 11100018), the National Natural Science Foundation of China (NSFC) and RGC Joint Research Scheme (3171101014, N_CityU114/17), Health@InnoHK funding support from the Innovation Technology Commission of the Hong Kong SAR (CityU 9445909). This work was also supported by City University of Hong Kong Neuroscience Research Infrastructure Grant (9610211) and Centre for Biosystems, Neuroscience, and Nanotechnology Grant (9360148).

## Supplementary Material

Supplementary_Figure_legend_kvae010

Review_file_OXFNSC-2023-019_R2_kvae010

## Data Availability

All data are provided within the manuscript and the supplementary materials.

## References

[1] Kofuji P , Araque A. Astrocytes and behavior. *Annu Rev Neurosci* 2021;44:49–67. 33406370 10.1146/annurev-neuro-101920-112225 PMC8257756

[2] Morelli M , Casagrande M, Forte G. Decision making: a theoretical review. *Integr Psychol Behav Sci* 2022;56:609–29. 34780011 10.1007/s12124-021-09669-x

[3] Paulus MP . Decision-making dysfunctions in psychiatry—altered homeostatic processing? *Science* 2007;318:602–6. 17962553 10.1126/science.1142997

[4] Cáceda R , Nemeroff CB, Harvey PD. Toward an understanding of decision making in severe mental illness. *J Neuropsychiatry Clin Neurosci* 2014;26:196–213. 24599051 10.1176/appi.neuropsych.12110268

[5] Bechara A , Damasio AR, Damasio H et al. Insensitivity to future consequences following damage to human prefrontal cortex. *Cognition* 1994;50:7–15. 8039375 10.1016/0010-0277(94)90018-3

[6] Rivalan M , Ahmed SH, Dellu-Hagedorn F. Risk-prone individuals prefer the wrong options on a rat version of the Iowa gambling task. *Biol Psychiatry* 2009;66:743–9. 19482266 10.1016/j.biopsych.2009.04.008

[7] Bush G , Vogt BA, Holmes J et al. Dorsal anterior cingulate cortex: a role in reward-based decision making. *Proc Natl Acad Sci* 2002;99:523–8. 11756669 10.1073/pnas.012470999 PMC117593

[8] Kennerley SW , Walton ME, Behrens TEJ et al. Optimal decision making and the anterior cingulate cortex. *Nat Neurosci* 2006;9:940–7. 16783368 10.1038/nn1724

[9] Mu L , Wang J, Cao B et al. Impairment of cognitive function by chemotherapy: association with the disruption of phase-locking and synchronization in anterior cingulate cortex. *Mol Brain* 2015;8:32. 26001812 10.1186/s13041-015-0125-y PMC4490721

[10] Allen NJ , Eroglu C. Cell biology of astrocyte-synapse interactions. *Neuron* 2017;96:697–708. 29096081 10.1016/j.neuron.2017.09.056 PMC5687890

[11] Doron A , Rubin A, Benmelech-Chovav A et al. Hippocampal astrocytes encode reward location. *Nature* 2022;609:772–8. 36045289 10.1038/s41586-022-05146-6

[12] Halassa MM , Haydon PG. Integrated brain circuits: astrocytic networks modulate neuronal activity and behavior. *Annu Rev Physiol* 2010;72:335–55. 20148679 10.1146/annurev-physiol-021909-135843 PMC3117429

[13] Kofuji P , Araque A. G-protein-coupled receptors in astrocyte-neuron communication. *Neuroscience* 2021;456:71–84. 32224231 10.1016/j.neuroscience.2020.03.025 PMC8817509

[14] Ota Y , Zanetti AT, Hallock RM. The role of astrocytes in the regulation of synaptic plasticity and memory formation. *Neural Plast* 2013;2013:185463. 24369508 10.1155/2013/185463 PMC3867861

[15] Bang J , Kim HY, Lee H. Optogenetic and chemogenetic approaches for studying astrocytes and gliotransmitters. *Exp Neurobiol* 2016;25:205–21. 27790055 10.5607/en.2016.25.5.205 PMC5081467

[16] Urban DJ , Roth BL. DREADDs (designer receptors exclusively activated by designer drugs): chemogenetic tools with therapeutic utility. *Annu Rev Pharmacol Toxicol* 2015;55:399–417. 25292433 10.1146/annurev-pharmtox-010814-124803

[17] Kol A , Adamsky A, Groysman M et al. Astrocytes contribute to remote memory formation by modulating hippocampal-cortical communication during learning. *Nat Neurosci* 2020;23:1229–39. 32747787 10.1038/s41593-020-0679-6 PMC7611962

[18] Akter M , Hasan M, Ramkrishnan AS et al. Astrocyte and L-lactate in the anterior cingulate cortex modulate schema memory and neuronal mitochondrial biogenesis. *eLife* 2023;12:e85751. 37960975 10.7554/eLife.85751 PMC10645423

[19] Iwai Y , Ozawa K, Yahagi K et al. Transient astrocytic Gq signaling underlies remote memory enhancement. *Front Neural Circuits* 2021;15:658343. 33828463 10.3389/fncir.2021.658343 PMC8019746

[20] Nagai J , Bellafard A, Qu Z et al. Specific and behaviorally consequential astrocyte G(q) GPCR signaling attenuation in vivo with iβARK. *Neuron* 2021;109:2256–2274.e9. 34139149 10.1016/j.neuron.2021.05.023 PMC8418870

[21] Wang J , Tu J, Cao B et al. Astrocytic l-lactate signaling facilitates amygdala-anterior cingulate cortex synchrony and decision making in rats. *Cell Rep* 2017;21:2407–18. 29186680 10.1016/j.celrep.2017.11.012

[22] Magistretti PJ , Allaman I. Lactate in the brain: from metabolic end-product to signalling molecule. *Nat Rev Neurosci* 2018;19:235–49. 29515192 10.1038/nrn.2018.19

[23] Suzuki A , Stern SA, Bozdagi O et al. Astrocyte-neuron lactate transport is required for long-term memory formation. *Cell* 2011;144:810–23. 21376239 10.1016/j.cell.2011.02.018 PMC3073831

[24] Vezzoli E , Cali C, De Roo M et al. Ultrastructural evidence for a role of astrocytes and glycogen-derived lactate in learning-dependent synaptic stabilization. *Cereb Cortex* 2020;30:2114–27. 31807747 10.1093/cercor/bhz226 PMC7174989

[25] Van Den Herrewegen Y , Sanderson TM, Sahu S et al. Side-by-side comparison of the effects of Gq- and Gi-DREADD-mediated astrocyte modulation on intracellular calcium dynamics and synaptic plasticity in the hippocampal CA1. *Mol Brain* 2021;14:144. 34544455 10.1186/s13041-021-00856-w PMC8451082

[26] Horvat A , Muhic M, Smolic T et al. Ca(2+) as the prime trigger of aerobic glycolysis in astrocytes. *Cell Calcium* 2021;95:102368. 33621899 10.1016/j.ceca.2021.102368

[27] Armbruster BN , Li X, Pausch MH et al. Evolving the lock to fit the key to create a family of G protein-coupled receptors potently activated by an inert ligand. *Proc Natl Acad Sci USA* 2007;104:5163–8. 17360345 10.1073/pnas.0700293104 PMC1829280

[28] Rivalan M , Valton V, Series P et al. Elucidating poor decision-making in a rat gambling task. *PLoS One* 2013;8:e82052. 24339988 10.1371/journal.pone.0082052 PMC3855331

[29] Pereira A Jr , Furlan FA. Astrocytes and human cognition: modeling information integration and modulation of neuronal activity. *Prog Neurobiol* 2010;92:405–20. 20633599 10.1016/j.pneurobio.2010.07.001

[30] Santello M , Toni N, Volterra A. Astrocyte function from information processing to cognition and cognitive impairment. *Nat Neurosci* 2019;22:154–66. 30664773 10.1038/s41593-018-0325-8

[31] Nam MH , Han KS, Lee J et al. Activation of astrocytic μ-opioid receptor causes conditioned place preference. *Cell Rep* 2019;28:1154–1166.e5. 31365861 10.1016/j.celrep.2019.06.071

[32] Liu S , Wong HY, Xie L et al. Astrocytes in CA1 modulate schema establishment in the hippocampal-cortical neuron network. *BMC Biol* 2022;20:250. 36352395 10.1186/s12915-022-01445-6 PMC9648012

[33] Jones ME , Paniccia JE, Lebonville CL et al. Chemogenetic manipulation of dorsal hippocampal astrocytes protects against the development of stress-enhanced fear learning. *Neuroscience* 2018;388:45–56. 30030056 10.1016/j.neuroscience.2018.07.015

[34] Yu X , Nagai J, Marti-Solano M et al. Context-specific striatal astrocyte molecular responses are phenotypically exploitable. *Neuron* 2020;108:1146–1162.e10. 33086039 10.1016/j.neuron.2020.09.021 PMC7813554

[35] Nagai J , Rajbhandari AK, Gangwani MR et al. Hyperactivity with disrupted attention by activation of an astrocyte synaptogenic cue. *Cell* 2019;177:1280–1292.e20. 31031006 10.1016/j.cell.2019.03.019 PMC6526045

[36] Vaidyanathan TV , Collard M, Yokoyama S et al. Cortical astrocytes independently regulate sleep depth and duration via separate GPCR pathways. *eLife* 2021;10:e63329. 33729913 10.7554/eLife.63329 PMC7968927

[37] Cai M , Wang H, Song H et al. Lactate is answerable for brain function and treating brain diseases: energy substrates and signal molecule. *Front Nutr* 2022;9:800901. 35571940 10.3389/fnut.2022.800901 PMC9099001

[38] Akter M , Li Y. Does astrocytic L-lactate enhance cognition through myelination? *Neural Regen Res* 2024;19:1167–8. 37905847 10.4103/1673-5374.385872 PMC11467931

[39] Dembitskaya Y , Piette C, Perez S et al. Lactate supply overtakes glucose when neural computational and cognitive loads scale up. *Proc Natl Acad Sci* 2022;119:e2212004119. 36375086 10.1073/pnas.2212004119 PMC9704697

[40] Iqbal Z , Liu S, Lei Z et al. Astrocyte L-lactate Signaling in the ACC regulates visceral pain aversive memory in rats. *Cells* 2023;12:26. 10.3390/cells12010026. PMC9818423 36611820

[41] Wang J , Zhang X, Cao B et al. Facilitation of synaptic transmission in the anterior cingulate cortex in viscerally hypersensitive rats. *Cereb Cortex* 2015;25:859–68. 24108805 10.1093/cercor/bht273 PMC4379994

[42] Zhou Z , Okamoto K, Onodera J et al. Astrocytic cAMP modulates memory via synaptic plasticity. *Proc Natl Acad Sci USA* 2021;118:e2016584118. 33452135 10.1073/pnas.2016584118 PMC7826339

[43] Scofield MD , Boger HA, Smith RJ et al. Gq-DREADD selectively initiates glial glutamate release and inhibits cue-induced cocaine seeking. *Biol Psychiatry* 2015;78:441–51. 25861696 10.1016/j.biopsych.2015.02.016 PMC4547911

[44] Mahmoud S , Gharagozloo M, Simard C et al. Astrocytes maintain glutamate homeostasis in the CNS by controlling the balance between glutamate uptake and release. *Cells* 2019;8:184. 10.3390/cells8020184. PMC6406900 30791579

[45] Pellerin L , Magistretti PJ. Glutamate uptake into astrocytes stimulates aerobic glycolysis: a mechanism coupling neuronal activity to glucose utilization. *Proc Natl Acad Sci USA* 1994;91:10625–9. 7938003 10.1073/pnas.91.22.10625 PMC45074

[46] Tarczyluk MA , Nagel DA, O'Neil JD et al. Functional astrocyte-neuron lactate shuttle in a human stem cell-derived neuronal network. *J Cereb Blood Flow Metab* 2013;33:1386–93. 23715062 10.1038/jcbfm.2013.81 PMC3764384

[47] Hasan M , Lei Z, Akter M et al. Chemogenetic activation of astrocytes promotes remyelination and restores cognitive deficits in visceral hypersensitive rats. *iScience* 2023;26:105840. 36619970 10.1016/j.isci.2022.105840 PMC9812719

[48] Hasan M , Kanna MS, Jun W et al. Schema-like learning and memory consolidation acting through myelination. *FASEB J* 2019;33:11758–75. 31366238 10.1096/fj.201900910R PMC6902718

[49] Xin W , Chan JR. Myelin plasticity: sculpting circuits in learning and memory. *Nat Rev Neurosci* 2020;21:682–94. 33046886 10.1038/s41583-020-00379-8 PMC8018611

[50] Ichihara Y , Doi T, Ryu Y et al. Oligodendrocyte progenitor cells directly utilize lactate for promoting cell cycling and differentiation. *J Cell Physiol* 2017;232:986–95. 27861886 10.1002/jcp.25690 PMC5299506

[51] Rinholm JE , Hamilton NB, Kessaris N et al. Regulation of oligodendrocyte development and myelination by glucose and lactate. *J Neurosci* 2011;31:538–48. 21228163 10.1523/JNEUROSCI.3516-10.2011 PMC3044866

[52] Hu X , Yu G, Liao X et al. Interactions between astrocytes and oligodendroglia in myelin development and related brain diseases. *Neurosci Bull* 2023;39:541–52. 36370324 10.1007/s12264-022-00981-z PMC10043111

[53] Narine M , Colognato H. Current insights into oligodendrocyte metabolism and its power to sculpt the myelin landscape. *Front Cell Neurosci* 2022;16:892968. 10.3389/fncel.2022.892968. PMC9097137 35573837

[54] Sanchez-Abarca LI , Tabernero A, Medina JM. Oligodendrocytes use lactate as a source of energy and as a precursor of lipids. *Glia* 2001;36:321–9. 11746769 10.1002/glia.1119

[55] Park J , Kim J, Mikami T. Exercise-induced lactate release mediates mitochondrial biogenesis in the hippocampus of mice via Monocarboxylate transporters. *Front Physiol* 2021;12:736905. 34603087 10.3389/fphys.2021.736905 PMC8481603

[56] Akter M , Ma H, Hasan M et al. Exogenous L-lactate administration in rat hippocampus increases expression of key regulators of mitochondrial biogenesis and antioxidant defense. *Front Mol Neurosci* 2023;16:1117146. 10.3389/fnmol.2023.1117146. PMC10062455 37008779

[57] Jacobs RA , Aboouf MA, Koester-Hegmann C et al. Erythropoietin promotes hippocampal mitochondrial function and enhances cognition in mice. *Commun Biol* 2021;4:938. 34354241 10.1038/s42003-021-02465-8 PMC8342552

[58] Gomez JL , Bonaventura J, Lesniak W et al. Chemogenetics revealed: DREADD occupancy and activation via converted clozapine. *Science* 2017;357:503–7. 28774929 10.1126/science.aan2475 PMC7309169

[59] Jendryka M , Palchaudhuri M, Ursu D et al. Pharmacokinetic and pharmacodynamic actions of clozapine-N-oxide, clozapine, and compound 21 in DREADD-based chemogenetics in mice. *Sci Rep* 2019;9:4522. 30872749 10.1038/s41598-019-41088-2 PMC6418145

[60] Mahler SV , Aston-Jones G. CNO evil? Considerations for the use of DREADDs in behavioral neuroscience. *Neuropsychopharmacology* 2018;43:934–6. 29303143 10.1038/npp.2017.299 PMC5854815

[61] Cao B , Wang J, Shahed M et al. Vagus nerve stimulation alters phase synchrony of the anterior cingulate cortex and facilitates decision making in rats. *Sci Rep* 2016;6:35135. 27731403 10.1038/srep35135 PMC5059720

