# Supplementary material for: Astrocytic GPCR signaling in the anterior cingulate cortex modulates decision making in rats
Source: Oxf Open Neurosci. 2024 Jun 22;3:kvae010. doi: 10.1093/oons/kvae010 (PMC11194462; doi:10.1093/oons/kvae010)
Supplement: Supplementary_Figure_legend_kvae010 [file supplementary_figure_legend_kvae010.docx]

**
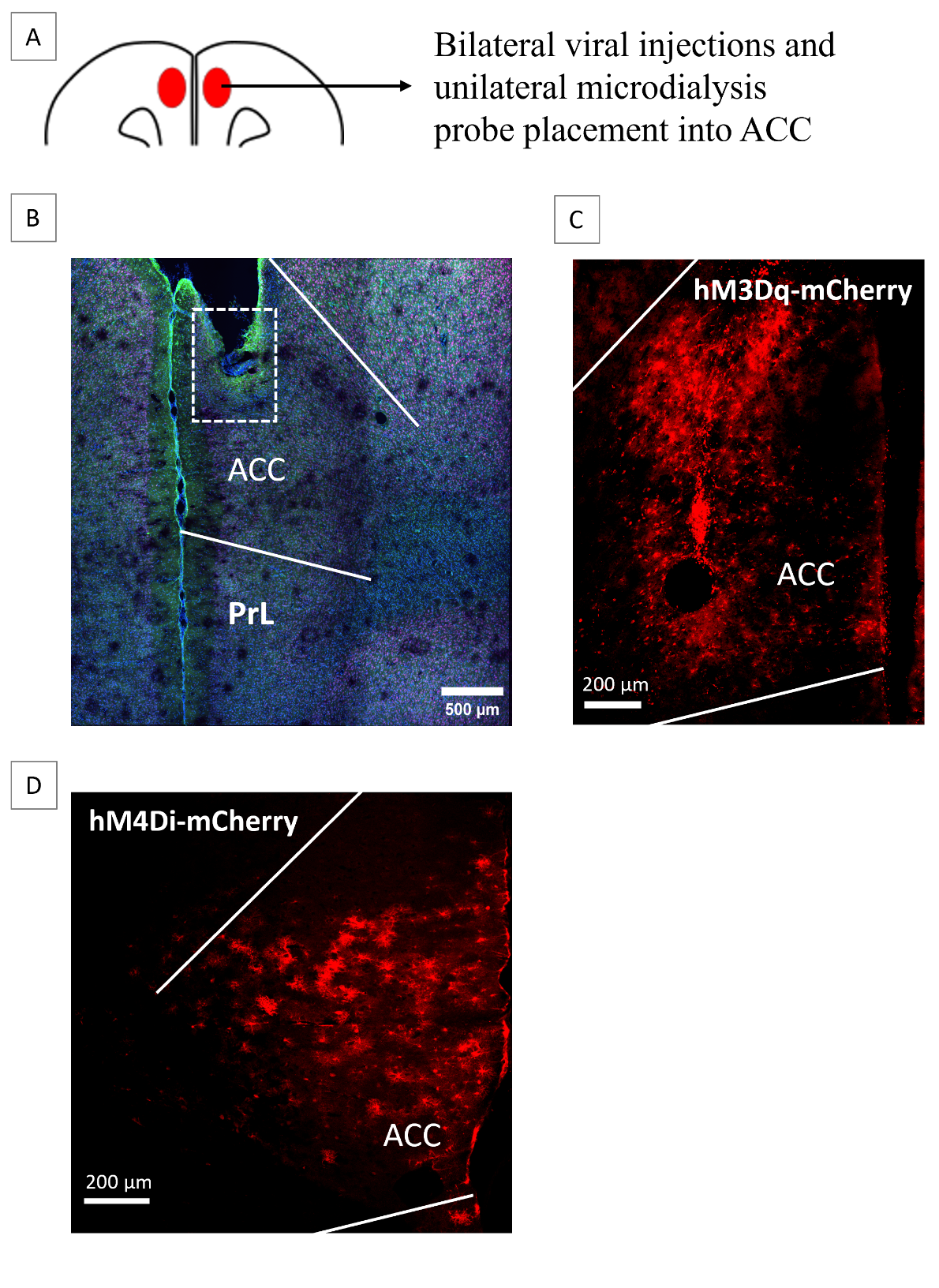
**

**Supplementary Fig. 1**

**A.** Schematic showing the location of bilateral viral injection into the ACC to express DREADDs and the location of microdialysis probe placement. **B.** Location of microdialysis probe placement (dashed box) in the ACC. **C.** Microdialysis probe location and the expression of hM3Dq-mCherry in the ACC. **D.** Expression of hM4Di-mCherry in the ACC.


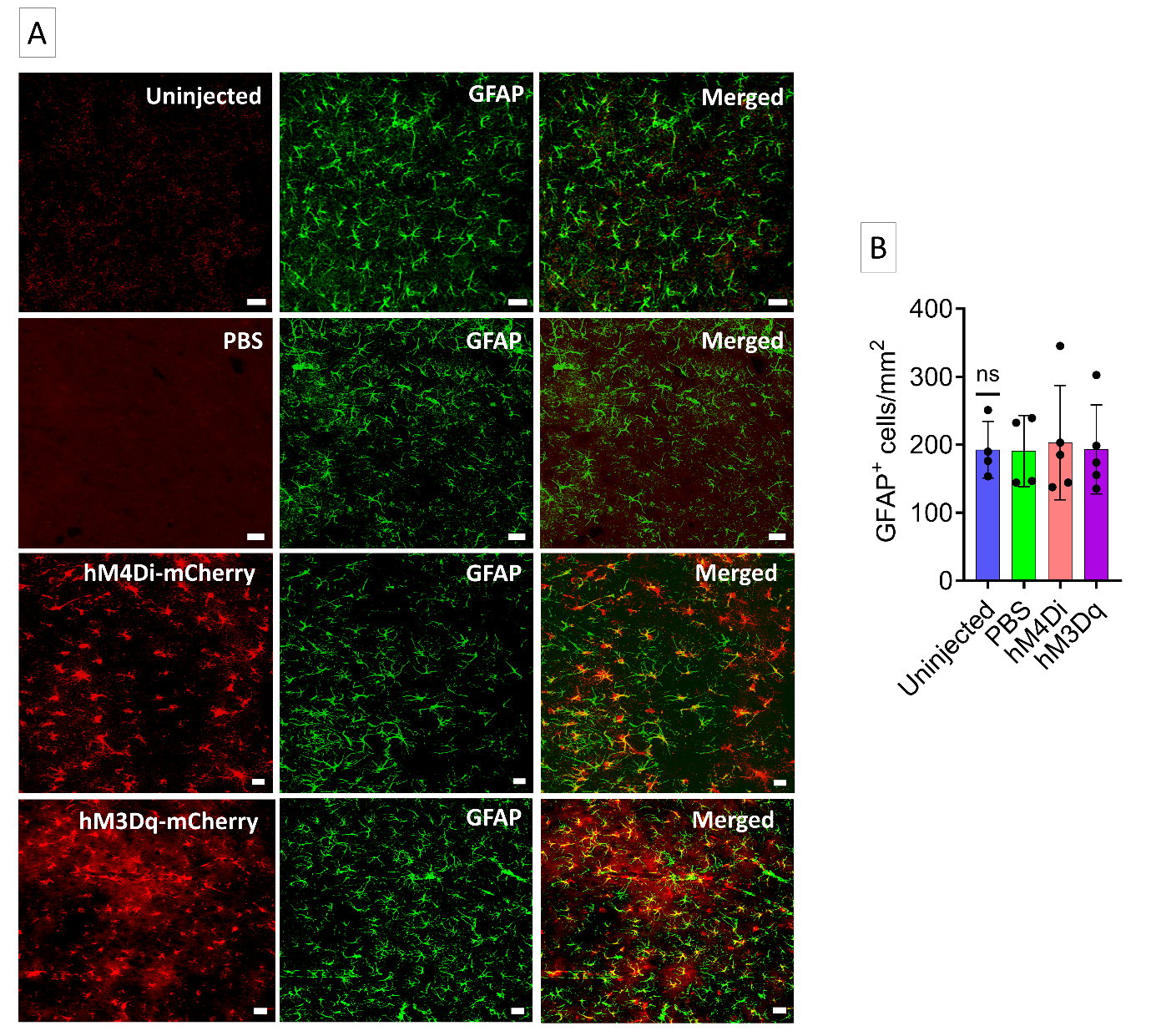


**Supplementary Fig. 2**

**A.** Representative confocal micrograph of DREADDs and GFAP in the ACC of uninjected, injected with PBS or AAV8-GFAP-hM4Di-mCherry or AAV8-GFAP-hM3Dq-mCherry group of rats. Scale bars: 20 µm. **B.** Number of GFAP^+^ cells/mm^2^ in the ACC of control rats (uninjected), control rats (injected with PBS), injected with AAV8-GFAP-hM4Di-mCherry or AAV8-GFAP-hM3Dq-mCherry (n=4 rats in uninjected, n=4 in PBS, n=5 in hM4Di, and n=5 in hM3Dq group). *p*=0.9908, One-Way ANOVA.
