# Supplementary material for: Astrocytic GPCR signaling in the anterior cingulate cortex modulates decision making in rats
Source: Oxf Open Neurosci. 2024 Jun 22;3:kvae010. doi: 10.1093/oons/kvae010 (PMC11194462; doi:10.1093/oons/kvae010)
Supplement: Review_file_OXFNSC-2023-019_R2_kvae010 [file review_file_oxfnsc-2023-019_r2_kvae010.zip › Review_file_OXFNSC-2023-019_R2_kvae010.odt]

Round 1 Reviews
Reviewer: 1

Comments to the Author
The authors used viral delivery methods to express hM3Dq and hM4Di in ACC astrocytes, aiming to activate the Gq and Gi signaling pathways, respectively for testing the roles of ACC astrocytes in decision making. They observed that Gq pathway activation in vivo within the ACC results in increased L-lactate levels, though the underlying mechanisms remain unidentified. The study also employs a modified version of the rat gambling task protocol, which appears less rigorously justified. In their analysis, the authors propose that the activation of Gq and Gi pathways produces contrasting effects. However, the molecular mechanisms behind these effects, and their relation to L-lactate mobilization under the two different activation conditions, are not clearly elucidated. The overall aim is fundamentally important, but there remain major concerns as elaborated below.

1. In Figure 1, the presented GFAP expression in the cortex appears atypical and too high, given a notably low presence of GFAP-positive astrocytes in rodent cortices. This observation raises concerns about astrogliosis due to potential overexpression of AAV-transduced DREADDs. The authors should include GFAP immunohistochemistry data in the absence of AAV injections for comparison.
2. To better understand the region-specific expression of astrocyte DREADDs in the ACC, please include low magnification images that clearly delineate this expression.
3. In Figure 4D, the pre-stimulation performance differences observed between groups (shown in red and green) suggest a potential sample bias, which might have led to a misinterpretation of the data. This aspect warrants further investigation. Otherwise, the data about Gq stimulation would be largely inconsequential.
4. Terminology clarification (Page 5), the terms "flexible" and "inflexible" used to describe decision-makers seem misleading, given that they are directly defined by the ≥70% preference criterion for advantageous choices used in this study and not related to flexibility. I recommend revising or removing these terms for clarity.
5. The study lacks a detailed exploration of the molecular mechanisms underlying the effects of CNO.
a. What makes difference between Gq- and Gi-stimulation? The accumulated literature indicates that both Gq and Gi-DREADD can induce astrocyte calcium signaling, which seems to contradict the authors' correlation between calcium, L-lactate production, and Gq signaling, as referenced from Horvat et al., 2021. This discrepancy needs addressing in discussion.
b. Furthermore, it would be insightful to understand how CNO administration during different learning phases (training, simple association learning, reversal learning) affects behavioral performances. This is fruitful to understand which cognitive domains are regulated by ACC astrocytes and temporal control with CNO that is a strong advantage of using DREADD system enable ones to study that. If difficult, please discuss.

Minor points:
1. In Figure 3, the design and methodology of the Rat Gambling Task (RGT) are not sufficiently detailed, particularly regarding training sessions, tasks, intervals in between, duration of each “phase”, and the timing post-surgery etc. Clarification of these aspects, along with a more explicit definition of Type-1 and Type-2 criteria that should be schematized, is necessary for a thorough understanding of the "six out of eight RGTs" statement. Additionally, the terms "good," "poor," and "worst" decision-makers require better definition to avoid confusion.
2. Page 7, Line 1: The term "intraperitoneal lactate injection into HPC" is confusing, as intraperitoneal injections are typically administered into the abdominal cavity, not directly into brain tissue.
3. Page 6, Line 3: Please correct the typographical error from "GCPR" to "GPCR."


Reviewer: 2

Comments to the Author
The paper describes the effects of manipulating astrocytic Gq and Gi signalling pathways on decision-making using the rodent gambling task. Rats were first trained on the rodent gambling task, then underwent to surgery for viral based expression of astrocytic Gq and Gi DREADD receptors in the anterior cingulate cortex. After a 3-week recovery, the effects of selectively activating astrocytic Gq receptors or Gi receptors on performance on the rodent gambling task was assessed. In “good” decision-makers, Gi pathway activation impaired decision-making, while in a subset of “poor” decision-makers Gq pathway activation improved decision-making.  Further, Gq pathway activation increased L-Lactate levels in the anterior cingulate cortex. It is speculated that the behavioral effects of Gi and Gq pathway activation are the result of changing L-lactate levels.

1. Although the paper demonstrates that the DREADD expression is limited to astrocytes with high penetrance, there is no discussion or illustration of the regional distribution of the DREADD receptors within the frontal cortex. A histological figure depicting the extent of DREADD expression in the ACC (and surrounding area) is warranted.  Similarly, a histological figure depicting the location of microdialysis probe placements within the ACC is warranted.
2. The sample size for several experiments is quite small (e.g., n=5 for the Type-1 Gi rats). Were power analyses conducted a priori to determine appropriate sample sizes? Low powered experiments run the risk of both failing to find statistically significant effects but also finding statistically significant effects that cannot be replicated. If a priori power analyses were not conducted, including the observed power of the statistical tests could be helpful.
3. In figure 4, the effects of ACC astrocytic Gi pathway inhibition and ACC astrocytic Gq pathway activation are depicted. Figures 4A and 4D depict advantageous decision-making on each of the RGT sessions from P1, P2, P3, P4 and P5.  I have several questions about how statistical analysis of these data were completed. Were % advantageous choices analyzed as a measure of decision-making? Based on my reading of the manuscript, this analysis was not conducted.  Why? It seems that this should be the primary measure of decision-making. Rather, it appears that the only measure of decision-making the number of food pellets earned (Figure 4B, FE)? Further, it is not clear whether the depicted number of food pellets earned reflects the average per session or the total across the 8 RGT sessions.
4. In figure 4D, the rats are divided into Type-1 and Type-2 decision makers.  This appears to be a post-hoc “exploratory” analysis rather than an a priori planned comparison. This should be reflected in the discussion. Further, the “combined” data should be presented since the original hypothesis was that Gq activation would improve decision-making.   Additionally, although the reason for the differential effects in Type-1 and Type-2 decision makers is not known, was there any exploration of histological differences in the two populations of rats? That is, was there similar expression of the DREADD receptors in the two groups?
5. Throughout the paper, it is indicated that Gi pathway activation impairs decision-making and that Gq pathway activation improves decision-making. It should be clarified that these effects are in specific populations of rodents—Gi pathway activation impairs decision-making in “advantageous” decision-makers while Gq pathway activation improves decision-making in “flexible” disadvantageous decision-makers.
6. CNO is known to have “off-target” effects and bind to many endogenous receptors in addition to DREADD receptors. Further, CNO can be reverse metabolized into clozapine and there is speculation that clozapine may be contributing to the observed behavioral effects of CNO. Because of these limitations, it is suggested that appropriate control groups be used (e.g., rats not expressing DREADD receptors but are administered CNO).  Given that such control groups were not utilized in the current experiment, can the authors reasonably attribute the observed effects to activation of DREADD receptors rather than just administration of CNO?  At least some discussion of the limitations of DREADD receptors and CNO is warranted. In particular it would be prudent to discuss how the design of the current experiment could assuage such concerns that the observed effects are due to CNO administration rather than selective activation of the specified signalling pathway.
7. Throughout the paper M. Akter et al., 2023 is referenced. When the reader looks to the reference list, they find there are 3 citations for M. Akter et al., 2023 and it is not clear which citation is appropriate in that instance.  Please clarify which reference is being cited, perhaps using letters, in the main body of the text.
 

Round 1 Decision Letter
Thank you for submitting manuscript ID OXFNSC-2023-019 entitled "Astrocytic GPCR signaling in the anterior cingulate cortex modulates decision making in rats" to Oxford Open Neuroscience.

Your manuscript has been seen by two reviewers with relevant expertise and I have read it myself.

Both reviewers find this work of interest, as do I, but they also point to several issues that should be addressed as well as requesting that you include additional data and images.

I am therefore inviting you to submit a revision that responds to these reviewer comments. The reviews are both very clear so I will not repeat their points here. When you submit your revision, please include a cover letter with your point-by-point responses to the reviewer comments, detailing any changes you have made to the manuscript.

I invite you to respond to the reviewer(s)' comments and revise your manuscript within the next 60 days from today date.

Please note that this journal operates with transparent peer review. This means that if your submission is accepted for publication, the full peer review history of your article will publish online alongside your article. This includes reviewer comments, editor decision letters, and your author responses.

To revise your manuscript, log into https://mc.manuscriptcentral.com/oxfnsc and enter your Author Centre, where you will find your manuscript title listed under "Manuscripts with Decisions."  Under "Actions," click on "Create a Revision."  Your manuscript number has been appended to denote a revision.

You may also click the below link to start the revision process (or continue the process if you have already started your revision) for your manuscript. If you use the below link you will not be required to login to ScholarOne Manuscripts.

*** PLEASE NOTE: This is a two-step process. After clicking on the link, you will be directed to a webpage to confirm. ***

https://mc.manuscriptcentral.com/oxfnsc?URL_MASK=9c821a2820e24ccb99f960fb08559765

You will be unable to make your revisions on the originally submitted version of the manuscript.  Instead, revise your manuscript using a word processing program and save it on your computer.  Please also highlight the changes to your manuscript within the document by using the track changes mode in MS Word or by using bold or colored text.

Once the revised manuscript is prepared, you can upload it and submit it through your Author Centre.

When submitting your revised manuscript, you will be able to respond to the comments made by the reviewer(s) in the space provided.  You can use this space to document any changes you make to the original manuscript.  In order to expedite the processing of the revised manuscript, please be as specific as possible in your response to the reviewer(s).

IMPORTANT:  Your original files are available to you when you upload your revised manuscript.  Please delete any redundant files before completing the submission.

Because we are trying to facilitate timely publication of manuscripts submitted to the Oxford Open Neuroscience, your revised manuscript should be uploaded within the next 60 days from today date.  If it is not possible for you to submit your revision in a reasonable amount of time, we may have to consider your paper as a new submission.

Once again, thank you for submitting your manuscript to the Oxford Open Neuroscience and I look forward to receiving your revision.

Response to Round 1 Reviews
Author response
We thank the reviewers and editor for their thoughtful comments and suggestions which has 
greatly improved our manuscript. Below is the point-by-point response to the reviewer’s 
comments.
Reviewer: 1
The authors used viral delivery methods to express hM3Dq and hM4Di in ACC astrocytes, 
aiming to activate the Gq and Gi signaling pathways, respectively for testing the roles of 
ACC astrocytes in decision making. They observed that Gq pathway activation in vivo within 
the ACC results in increased L-lactate levels, though the underlying mechanisms remain 
unidentified. The study also employs a modified version of the rat gambling task protocol, 
which appears less rigorously justified. In their analysis, the authors propose that the 
activation of Gq and Gi pathways produces contrasting effects. However, the molecular 
mechanisms behind these effects, and their relation to L-lactate mobilization under the two 
different activation conditions, are not clearly elucidated. The overall aim is fundamentally 
important, but there remain major concerns as elaborated below.
1. In Figure 1, the presented GFAP expression in the cortex appears atypical and too high, 
given a notably low presence of GFAP-positive astrocytes in rodent cortices. This 
observation raises concerns about astrogliosis due to potential overexpression of AAVtransduced DREADDs. The authors should include GFAP immunohistochemistry data in the 
absence of AAV injections for comparison.
In Supplementary Fig. 2 of the revised manuscript, we have now included data of GFAP 
immunohistochemistry of ACC of rats injected with PBS, or AAV8-GFAP-hM4Di-mCherry
(Gi), or AAV8-GFAP-hM3Dq-mCherry (Gq). We observed no significant difference in the 
three groups. The figure and legend is given below:
2
Supplementary Fig. 2 
A. Representative confocal micrograph of DREADDs and GFAP in the ACC of the 
mice injected with PBS or AAV8-GFAP-hM4Di-mCherry or AAV8-GFAP-hM3DqmCherry. Scale bars: 20 µm. B. Number of GFAP+
cells/mm2
in the ACC of rats 
injected with PBS or AAV8-GFAP-hM4Di-mCherry or AAV8-GFAP-hM3DqmCherry (n=4 rats in PBS, n=5 in hM4Di, and n=5 in hM3Dq group). ns=not 
significant, unpaired Student’s t-test.
2. To better understand the region-specific expression of astrocyte DREADDs in the ACC, 
please include low magnification images that clearly delineate this expression.
In Supplementary Fig. 1 of the revised manuscript, we have now added images of the regionspecific expression of Gi and Gq DREADDs in the ACC as given below:
 
 
 
 
 
 
 
 
 
 
 
3
Supplementary Fig. 1
A. Schematic showing the location of bilateral viral injection into the ACC to express 
DREADDs and the location of microdialysis probe placement. B. Location of 
microdialysis probe placement (dashed box) in the ACC. C. Microdialysis probe 
location and the expression of hM3Dq-mCherry in the ACC. D. Expression of 
hM4Di-mCherry in the ACC.
3. In Figure 4D, the pre-stimulation performance differences observed between groups 
(shown in red and green) suggest a potential sample bias, which might have led to a 
misinterpretation of the data. This aspect warrants further investigation. Otherwise, the data 
about Gq stimulation would be largely inconsequential.
After we divided the rats into two groups based on whether they were consistently good 
decision maker or not (i.e., Type-1 = good decision maker in ≥6 RGT sessions out of 8 
sessions in Phase-1; Type-2 = good decision maker in <6 out of 8 RGT sessions in Phase-1), 
4
all the Type-2 rats (n=17) were assigned to the experimental paradigm of Phase-2 to Phase-5 
shown in Figure 4E (of revised manuscript). In phase-3, all these Type-2 rats received CNO 
to stimulate Gq pathway. The subgrouping shown in Figure 4E is based on the result from 
Phase-3 which was done after the experiment was completed. The subgrouping is therefore 
retrospective based on whether or not they became consistently good decision maker in 
Phase-3 (i.e., good decision making in ≥6 RGT sessions out of 8 sessions in Phase-3). The 
observed pre-stimulation performance difference of Phase-1 was evident after this 
retrospective analysis, and shows that the rats that became consistently good decision makers
(i.e. became Type-1) in Phase-3 already showed more advantageous choices in Phase-1
compared to the other rats (also shown in Fig. 4G). However, to further clarify, we have now 
included the combined data in the Figure 4E of the revised manuscript, which shows no 
difference in the decision making between Phase-1 and Phase-3 for the whole cohort. We 
have further clarified this and mentioned in the result section of the revised manuscript (given 
below) highlighting that we observed improved advantageous choices in only a subset of rats, 
not in the whole cohort. 
… we injected AAV8-GFAP-hM3Dq-mCherry bilaterally into the ACC to express 
hM3Dq in the ACC astrocytes of Type-2 rats (n=17) identified in Phase-1. In Phase-3, 
they all received CNO (3 mg/kg body weight, I.P.) before 15 mins of each RGT 
session to activate the Gq signaling pathway in the ACC astrocytes (Fig. 4E). There 
was no significant improvement in the advantageous choices percentage in the whole 
cohort data of these Type-2 rats (Fig. 4E and 4F) as well as no differences in the food 
pellets awarded and nose pokes/min (Fig. 4I and 4J). However, when we sub-grouped 
the cohort based on whether the individual rats showed improved decision making 
defined by becoming consistently good decision maker in Phase-3 (i.e., had good 
decision making in ≥6 RGTs in the 8 sessions of Phase-3), we found that 41.2% 
(7/17) of the rats became consistently good decision makers in Phase-3 (Fig. 4E and 
4G)….
5
Fig. 4 | Astrocytic Gi pathway activation in ACC impairs decision making in 
RGTs whereas Gq pathway activation can improve decision making in a 
subgroup of rats
A-D. Effect of ACC astrocytic Gi pathway activation in decision making. A. Effect of 
ACC astrocytic Gi pathway activation in Type-1 rats identified in Phase-1 (n=5). 
Astrocytic Gi activation with I.P. CNO administration dramatically impaired decision 
making in Phase-3 whereas the impairment was abolished in Phase-4 where I.P. saline 
was given. The percentage of advantageous choices remained unchanged despite 
reversal of advantageous choices in Phase-5. B. Mean advantageous choices 
percentage per session for the Type-1 rats (n=5) during Phase-1, Phase-3, and Phase4. ***p<0.001, ****p<0.0001, paired Student’s t-test, observed power for Phase-1 vs. 
3 and Phase-3 vs 4 is >0.99 and >0.99, respectively. C. Mean food pellets per session 
6
obtained by the Type-1 rats (n=5) during Phase-1 and Phase-3. *p<0.05, paired 
Student’s t-test. D. Mean nose pokes per minute of Type-1 rats (n=5) during Phase-1 
and Phase-3. ns=not significant, paired Student’s t-test.
E-J. Effect of ACC astrocytic Gq pathway activation in decision making. E. Effect of 
ACC astrocytic Gq pathway activation in Type-2 rats identified in Phase-1 (n=17). 
Seven rats (41.2%) turned into Type-1 rats (i.e., good decision making in ≥6 RGTs) in 
Phase-3 where astrocytic Gq pathway was activated with I.P. CNO administration. 
They turned back into Type-2 (i.e., good decision making in <6 RGTs) in Phase-4 
where I.P. saline was given. The percentage of advantageous choices remained 
similarly poor during reversal of advantageous choices in Phase-5. Other rats (58.5%) 
remained as Type-2 in Phase-3. F-H. Mean advantageous choices percentage per 
session during Phase-1, Phase-3, and Phase-4 for the whole cohort of Type-2 rats 
(n=17), Type-2 rats turned into Type-1 in Phase-3 (n=7), and Type-2 rats remained as 
Type-2 in Phase-3, respectively. **p<0.01, ***p<0.001, ****p<0.0001, ns=not 
significant, paired Student’s t-test, observed power for Phase-1 vs. 3 and Phase-3 vs 4 
is 0.07 and >0.99 (F), 0.99 and >0.99 (G), 0.89 and 0.65 (H), respectively. I. Mean 
food pellets per session obtained by the Type-2 rats during Phase-1 and Phase-3. 
*p<0.05, ns=not significant, paired Student’s t-test. J. Mean nose pokes per minute of 
Type-2 rats during Phase-1 and Phase-3. ns=not significant, paired Student’s t-test.
4. Terminology clarification (Page 5), the terms "flexible" and "inflexible" used to describe 
decision-makers seem misleading, given that they are directly defined by the ≥70% 
preference criterion for advantageous choices used in this study and not related to flexibility. 
I recommend revising or removing these terms for clarity.
We have removed the terms “flexible” and “inflexible”. 
5. The study lacks a detailed exploration of the molecular mechanisms underlying the effects 
of CNO.
a. What makes difference between Gq- and Gi-stimulation? The accumulated literature 
indicates that both Gq and Gi-DREADD can induce astrocyte calcium signaling, which 
seems to contradict the authors' correlation between calcium, L-lactate production, and Gq 
signaling, as referenced from Horvat et al., 2021. This discrepancy needs addressing in 
discussion.
We have further clarified this aspect in the discussion section of the revised manuscript as 
follows:
We recently demonstrated that astrocytic Gi pathway activation in the ACC impairs 
paired associates learning and schema memory formation by decreasing L-lactate 
level in the ACC ECF which was associated with a decrease in the astrocytic cAMP 
upon Gi activation (Akter et al., 2023). Furthermore, we previously reported impaired 
7
decision making in visceral hypersensitive rats resulting from a decrease in ACC Llactate levels (Wang et al., 2015; Wang et al., 2017). Therefore, we speculate that the 
impaired decision making observed upon astrocytic Gi activation in our current study 
is mediated by a decrease in L-lactate level in the ACC. In this study, we also found 
that astrocytic Gq pathway activation increases L-lactate level in the ACC. Although 
the detailed mechanisms by which astrocytic GPCRs regulate L-lactate level are yet to 
be elucidated, it is well established that astrocytic Gq activation results in a consistent 
increase in spontaneous astrocytic Ca2+ events (Van Den Herrewegen et al., 2021), 
whereas, astrocytic Gi activation induces a decrease in cAMP (Akter et al., 2023) and 
a transient rise followed by a decrease in intracellular Ca2+ (Kol et al., 2020). 
Activation of hippocampal astrocytic cAMP enhanced synaptic plasticity and memory 
by increasing release of L-lactate from astrocytes (Zhou et al., 2021). Another study 
demonstrated that Ca2+ signals are key triggers for aerobic glycolysis in astrocytes 
resulting in increased L-lactate production and this process can be augmented by 
cAMP (Horvat et al., 2021). Based on this accumulated evidence, it is tempting to 
speculate that the decrease in cAMP upon astrocytic Gi activation and the increase in 
Ca2+ events upon astrocytic Gq activation are the likely mediators of the decrease and 
increase in L-lactate levels in the ACC ECF, respectively.
b. Furthermore, it would be insightful to understand how CNO administration during 
different learning phases (training, simple association learning, reversal learning) affects 
behavioral performances. This is fruitful to understand which cognitive domains are 
regulated by ACC astrocytes and temporal control with CNO that is a strong advantage of 
using DREADD system enable ones to study that. If difficult, please discuss.
We have mentioned this limitation in the discussion section of the revised manuscript as 
follows:
While our current study design allowed us to observe the effect of Gi or Gq 
activation/inactivation through three key phases (Phase-1, Phase-3, and Phase-4) of 
RGTs, it would be further informative to investigate how different GPCR 
manipulations affect the decision making in longer terms when the advantageous 
choices are reversed (currently only 3 RGTs were done in Phase-5 where the 
advantageous positions were reversed). Moreover, we did not investigate how 
different GPCR manipulations during the RGT training (T1-T4) affect subsequent 
decision making in the RGT sessions. Further studies are needed to address these 
limitations.
Minor points:
1. In Figure 3, the design and methodology of the Rat Gambling Task (RGT) are not 
sufficiently detailed, particularly regarding training sessions, tasks, intervals in between, 
duration of each “phase”, and the timing post-surgery etc. Clarification of these aspects, 
along with a more explicit definition of Type-1 and Type-2 criteria that should be 
8
schematized, is necessary for a thorough understanding of the "six out of eight RGTs" 
statement. Additionally, the terms "good," "poor," and "worst" decision-makers require 
better definition to avoid confusion.
We have now provided further details in Fig. 3A along with several key definitions as 
follows:
2. Page 7, Line 1: The term "intraperitoneal lactate injection into HPC" is confusing, as 
intraperitoneal injections are typically administered into the abdominal cavity, not directly 
into brain tissue.
It is now corrected as follows:
Increased plasma lactate due to exercise or intraperitoneal lactate injection can 
promote mitochondrial biogenesis in the hippocampus of mice.
3. Page 6, Line 3: Please correct the typographical error from "GCPR" to "GPCR."
Corrected.
 
 
 
 ne nose poke one pellet reward
 Two nose pokes one pellet reward
 Two pellets rewarded for a choice
 ne pellet rewarded for a choice
 The RGT sessions of these phases were done 
with 8 hours intersession interval.
 ach RGT session is of one hour duration.
 Rat is defined as b 
 0 preference for the advantageous options 
in the last 0 minutes of RGT session.
 , , and Rat is defined as in 
a phase if it is a good decision maker in 6 out 
of 8 RGT sessions in that particular phase 
defined as if it is good decision maker 
in <6 out of 8 sessions.
3 weeks
15 da s
15 da s
15 da s
5 da s
3 da s
9
Reviewer: 2
The paper describes the effects of manipulating astrocytic Gq and Gi signalling pathways on 
decision-making using the rodent gambling task. Rats were first trained on the rodent 
gambling task, then underwent to surgery for viral based expression of astrocytic Gq and Gi 
DREADD receptors in the anterior cingulate cortex. After a 3-week recovery, the effects of 
selectively activating astrocytic Gq receptors or Gi receptors on performance on the rodent 
gambling task was assessed. In “good” decision-makers, Gi pathway activation impaired 
decision-making, while in a subset of “poor” decision-makers Gq pathway activation 
improved decision-making. Further, Gq pathway activation increased L-Lactate levels in the 
anterior cingulate cortex. It is speculated that the behavioral effects of Gi and Gq pathway 
activation are the result of changing L-lactate levels.
1. Although the paper demonstrates that the DREADD expression is limited to astrocytes with 
high penetrance, there is no discussion or illustration of the regional distribution of the 
DREADD receptors within the frontal cortex. A histological figure depicting the extent of 
DREADD expression in the ACC (and surrounding area) is warranted. Similarly, a 
histological figure depicting the location of microdialysis probe placements within the ACC 
is warranted.
In Supplementary Fig. 1 of the revised manuscript, we have now added images of the 
location of microdialysis probe and the region-specific expression of Gi and Gq DREADDs 
in the ACC as given below:
10
Supplementary Fig. 1
A. Schematic showing the location of bilateral viral injection into the ACC to express 
DREADDs and the location of microdialysis probe placement. B. Location of 
microdialysis probe placement (dashed box) in the ACC. C. Microdialysis probe 
location and the expression of hM3Dq-mCherry in the ACC. D. Expression of 
hM4Di-mCherry in the ACC.
2. The sample size for several experiments is quite small (e.g., n=5 for the Type-1 Gi rats). 
Were power analyses conducted a priori to determine appropriate sample sizes? Low 
powered experiments run the risk of both failing to find statistically significant effects but 
also finding statistically significant effects that cannot be replicated. If a priori power 
analyses were not conducted, including the observed power of the statistical tests could be 
helpful.
11
Priori power analyses were not conducted. In the revised manuscript, we have now provided 
the observed power of the statistical tests in the legend of Fig. 4B,F,G,H.
Fig. 4 | Astrocytic Gi pathway activation in ACC impairs decision making in 
RGTs whereas Gq pathway activation can improve decision making in a 
subgroup of rats
A-D. Effect of ACC astrocytic Gi pathway activation in decision making. A. Effect of 
ACC astrocytic Gi pathway activation in Type-1 rats identified in Phase-1 (n=5). 
Astrocytic Gi activation with I.P. CNO administration dramatically impaired decision 
making in Phase-3 whereas the impairment was abolished in Phase-4 where I.P. saline 
was given. The percentage of advantageous choices remained unchanged despite 
reversal of advantageous choices in Phase-5. B. Mean advantageous choices 
percentage per session for the Type-1 rats (n=5) during Phase-1, Phase-3, and Phase-
 
 
 
 
 
 
12
4. ***p<0.001, ****p<0.0001, paired Student’s t-test, observed power for Phase-1 vs. 
3 and Phase-3 vs 4 is >0.99 and >0.99, respectively. C. Mean food pellets per session 
obtained by the Type-1 rats (n=5) during Phase-1 and Phase-3. *p<0.05, paired 
Student’s t-test. D. Mean nose pokes per minute of Type-1 rats (n=5) during Phase-1 
and Phase-3. ns=not significant, paired Student’s t-test.
E-J. Effect of ACC astrocytic Gq pathway activation in decision making. E. Effect of 
ACC astrocytic Gq pathway activation in Type-2 rats identified in Phase-1 (n=17). 
Seven rats (41.2%) turned into Type-1 rats (i.e., good decision making in ≥6 RGTs) in 
Phase-3 where astrocytic Gq pathway was activated with I.P. CNO administration. 
They turned back into Type-2 (i.e., good decision making in <6 RGTs) in Phase-4 
where I.P. saline was given. The percentage of advantageous choices remained 
similarly poor during reversal of advantageous choices in Phase-5. Other rats (58.5%) 
remained as Type-2 in Phase-3. F-H. Mean advantageous choices percentage per 
session during Phase-1, Phase-3, and Phase-4 for the whole cohort of Type-2 rats 
(n=17), Type-2 rats turned into Type-1 in Phase-3 (n=7), and Type-2 rats remained as 
Type-2 in Phase-3, respectively. **p<0.01, ***p<0.001, ****p<0.0001, ns=not 
significant, paired Student’s t-test, observed power for Phase-1 vs. 3 and Phase-3 vs 4 
is 0.07 and >0.99 (F), 0.99 and >0.99 (G), 0.89 and 0.65 (H), respectively. I. Mean 
food pellets per session obtained by the Type-2 rats during Phase-1 and Phase-3. 
*p<0.05, ns=not significant, paired Student’s t-test. J. Mean nose pokes per minute of 
Type-2 rats during Phase-1 and Phase-3. ns=not significant, paired Student’s t-test.
3. In figure 4, the effects of ACC astrocytic Gi pathway inhibition and ACC astrocytic Gq 
pathway activation are depicted. Figures 4A and 4D depict advantageous decision-making 
on each of the RGT sessions from P1, P2, P3, P4 and P5. I have several questions about how 
statistical analysis of these data were completed. Were % advantageous choices analyzed as 
a measure of decision-making? Based on my reading of the manuscript, this analysis was not 
conducted. Why? It seems that this should be the primary measure of decision-making. 
Rather, it appears that the only measure of decision-making the number of food pellets 
earned (Figure 4B, FE)? Further, it is not clear whether the depicted number of food pellets 
earned reflects the average per session or the total across the 8 RGT sessions.
Indeed the primary measure of decision-making was the % advantageous choices. In every 
RGT session, each rats was assessed for % advantageous choices in the last 20 mins of the 60 
mins session. If the % advantageous choice was ≥ 0 , it was considered as good decision 
maker in that particular RGT session. Then for Phase-1, 3, or 4 (each of which consisted of 8 
RGT sessions), if a rat showed good decision making in ≥6 out the 8 RGT sessions, it was 
considered as Type-1 rat (i.e., a consistently good decision-maker) rat in that particular phase.
The results that were shown in Fig. 4A represents the mean ± SD of the advantageous choices 
for 5 Type-1 rats identified in Phase-1. The results that were shown in Fig. 4D (Fig. 4E of the 
revised manuscript) represents the mean ± SD of the advantageous choices for the Type-2
rats identified in Phase-1. To further clarify in the revised manuscript, we have added new 
Fig. 4B,F,G,H (figures attached tin the response to the previous comment) to present the 
13
mean % advantageous choices in the 8 RGT sessions of the three main phases (P1, P3, and 
P4). These figures further summarizes the % of advantageous choices data of these rats. The 
statistical test results (paired t test) is provided in the figure legend. 
The number of food pellets earned reflects the average per session which is now clarified in 
the figure legend.
4. In figure 4D, the rats are divided into Type-1 and Type-2 decision makers. This appears to 
be a post-hoc “exploratory” analysis rather than an a priori planned comparison. This 
should be reflected in the discussion. Further, the “combined” data should be presented 
since the original hypothesis was that Gq activation would improve decision-making. 
Additionally, although the reason for the differential effects in Type-1 and Type-2 decision 
makers is not known, was there any exploration of histological differences in the two 
populations of rats? That is, was there similar expression of the DREADD receptors in the 
two groups?
Yes, it is post-hoc exploratory analysis based on the categorization of rats from the 
advantageous choices in the RGT sessions of Phase-3. In Fig. 4 of the revised manuscript, we 
have now included the combined data (Fig. 4E,F,I,J). We have also clarified it in the result 
section as follows:
… we injected AAV8-GFAP-hM3Dq-mCherry bilaterally into the ACC to express 
hM3Dq in the ACC astrocytes of Type-2 rats (n=17) identified in Phase-1. In Phase-3, 
they all received CNO (3 mg/kg body weight, I.P.) before 15 mins of each RGT 
session to activate the Gq signaling pathway in the ACC astrocytes (Fig. 4E). There 
was no significant improvement in the advantageous choices percentage in the whole 
cohort data of these Type-2 rats (Fig. 4E and 4F) as well as no differences in the food 
pellets awarded and nose pokes/min (Fig. 4I and 4J). However, when we sub-grouped 
the cohort based on whether the individual rats showed improved decision making 
defined by becoming consistently good decision maker in Phase-3 (i.e., had good 
decision making in ≥6 RGTs in the 8 sessions of Phase-3), we found that 41.2% 
(7/17) of the rats became consistently good decision makers in Phase-3 (Fig. 4E and 
 G)….
While we acknowledge that the exploration of whether there is differential expression of 
DREADD in these two types of rats is of importance, we did not conduct this analysis. We 
have acknowledged this limitation in the discussion as follows:
… it remains an open question whether the enhanced decision making observed upon 
astrocytic Gq activation in a subgroup of disadvantageous decision-making rats, 
might have been mediated by enhanced myelination in the ACC due to increased Llactate level. It is also unclear why the other rats did not show improvement in the 
advantageous choice preference. One limitation of our current study is that we did not 
14
exclude the possibility of differential expression of hM3Dq in these two types of rats. 
Further studies are needed to better understand whether these results represent true 
biological variations among animals or due to difference in the expression level of the 
hM3Dq.
5. Throughout the paper, it is indicated that Gi pathway activation impairs decision-making 
and that Gq pathway activation improves decision-making. It should be clarified that these 
effects are in specific populations of rodents—Gi pathway activation impairs decisionmaking in “advantageous” decision-makers while Gq pathway activation improves decisionmaking in “flexible” disadvantageous decision-makers.
We have revised the manuscript accordingly.
6. CNO is known to have “off-target” effects and bind to many endogenous receptors in 
addition to DREADD receptors. Further, CNO can be reverse metabolized into clozapine and 
there is speculation that clozapine may be contributing to the observed behavioral effects of 
CNO. Because of these limitations, it is suggested that appropriate control groups be used 
(e.g., rats not expressing DREADD receptors but are administered CNO). Given that such 
control groups were not utilized in the current experiment, can the authors reasonably 
attribute the observed effects to activation of DREADD receptors rather than just 
administration of CNO? At least some discussion of the limitations of DREADD receptors 
and CNO is warranted. In particular it would be prudent to discuss how the design of the 
current experiment could assuage such concerns that the observed effects are due to CNO 
administration rather than selective activation of the specified signalling pathway.
We have discussed this aspect in the discussion section of the revised manuscript as follows:
One important consideration while interpreting results obtained with DREADD and 
CNO system is the potential off-target effects of CNO. Gomez et. al. investigated the 
mechanism of action of CNO in DREADD-expressing animals (Gomez et al., 2017). 
They found that upon systemic injection of CNO it is converted into clozapine that 
crosses the blood-brain barrier and binds to DREADDs expressed in the central 
nervous system. The study showed that I.P. injection of 0.1 mg/kg clozapine (which is 
equivalent to 10 mg/kg CNO) decreases locomotor activity only in the hM4Diexpressing (in basal forebrain) rats but not in control rats without hM4Di expression. 
In our study, we used 3 mg/kg I.P. CNO (expected to be equivalent to 0.03 mg/kg 
clozapine) which is below the level at which the study did not find DREADDindependent effect. Furthermore, although in the current study we did not use a 
control group to investigate if CNO alone has off-target effects, our previous study 
using the same dose showed that it does not affect the paired-associate learning and 
schema memory formation as well as the general activity in rats (Akter et al., 2023). 
This is also consistent with other reports which failed to find significant effects of up 
15
to 10 mg/kg CN on various motivated behaviors in non-DREADD-expressing 
animals, at least within a 30–150 min timeframe after I.P. injection (Mahler & AstonJones, 2018; Jendryka et al., 2019). Therefore, the effects observed in our current 
study is unlikely to be due to non-specific or off-target effects of CNO as we have 
used a dose of 3 mg/kg and the RGT sessions were started after 15 mins and lasted for 
only 60 mins.
7. Throughout the paper M. Akter et al., 2023 is referenced. When the reader looks to the 
reference list, they find there are 3 citations for M. Akter et al., 2023 and it is not clear which 
citation is appropriate in that instance. Please clarify which reference is being cited, 
perhaps using letters, in the main body of the text.
The citations and the reference list is updated and indicated with Akter et al., 2023a, Akter et 
al., 2023b, and Akter & Li, 2024.
References
Akter, M., Hasan, M., Ramkrishnan, A. S., Iqbal, Z., Zheng, X., Fu, Z., Lei, Z., Karim, A., & 
Li, Y. (2023). Astrocyte and L-lactate in the anterior cingulate cortex modulate 
schema memory and neuronal mitochondrial biogenesis. eLife, 12, e85751. 
https://doi.org/10.7554/eLife.85751
Gomez, J. L., Bonaventura, J., Lesniak, W., Mathews, W. B., Sysa-Shah, P., Rodriguez, L. 
A., Ellis, R. J., Richie, C. T., Harvey, B. K., Dannals, R. F., Pomper, M. G., Bonci, 
A., & Michaelides, M. (2017). Chemogenetics revealed: DREADD occupancy and 
activation via converted clozapine. Science, 357(6350), 503-507. 
https://doi.org/10.1126/science.aan2475
Horvat, A., Muhic, M., Smolic, T., Begic, E., Zorec, R., Kreft, M., & Vardjan, N. (2021). 
Ca(2+) as the prime trigger of aerobic glycolysis in astrocytes. Cell Calcium, 95, 
102368. https://doi.org/10.1016/j.ceca.2021.102368
Jendryka, M., Palchaudhuri, M., Ursu, D., van der Veen, B., Liss, B., Katzel, D., Nissen, W., 
& Pekcec, A. (2019). Pharmacokinetic and pharmacodynamic actions of clozapine-Noxide, clozapine, and compound 21 in DREADD-based chemogenetics in mice. Sci 
Rep, 9(1), 4522. https://doi.org/10.1038/s41598-019-41088-2
Kol, A., Adamsky, A., Groysman, M., Kreisel, T., London, M., & Goshen, I. (2020). 
Astrocytes contribute to remote memory formation by modulating hippocampalcortical communication during learning. Nat Neurosci, 23(10), 1229-1239. 
https://doi.org/10.1038/s41593-020-0679-6
Mahler, S. V., & Aston-Jones, G. (2018). CNO Evil? Considerations for the Use of 
DREADDs in Behavioral Neuroscience. Neuropsychopharmacology, 43(5), 934-936. 
https://doi.org/10.1038/npp.2017.299
16
Van Den Herrewegen, Y., Sanderson, T. M., Sahu, S., De Bundel, D., Bortolotto, Z. A., & 
Smolders, I. (2021). Side-by-side comparison of the effects of Gq- and Gi-DREADDmediated astrocyte modulation on intracellular calcium dynamics and synaptic 
plasticity in the hippocampal CA1. Mol Brain, 14(1), 144. 
https://doi.org/10.1186/s13041-021-00856-w
Wang, J., Tu, J., Cao, B., Mu, L., Yang, X., Cong, M., Ramkrishnan, A. S., Chan, R. H. M., 
Wang, L., & Li, Y. (2017). Astrocytic l-Lactate Signaling Facilitates AmygdalaAnterior Cingulate Cortex Synchrony and Decision Making in Rats. Cell Rep, 21(9), 
2407-2418. https://doi.org/10.1016/j.celrep.2017.11.012
Wang, J., Zhang, X., Cao, B., Liu, J., & Li, Y. (2015). Facilitation of synaptic transmission in 
the anterior cingulate cortex in viscerally hypersensitive rats. Cereb Cortex, 25(4), 
859-868. https://doi.org/10.1093/cercor/bht273
Zhou, Z., Okamoto, K., Onodera, J., Hiragi, T., Andoh, M., Ikawa, M., Tanaka, K. F., 
Ikegaya, Y., & Koyama, R. (2021). Astrocytic cAMP modulates memory via synaptic 
plasticity. Proc Natl Acad Sci U S A, 118(3), e2016584118. 
https://doi.org/10.1073/pnas.2016584118

Round 2 Reviews
Reviewer: 1

Comments to the Author
1. In Supplementary Figure 2, the authors should include intact brain tissue controls and conduct a comparison of GFAP levels across all four groups (intact, PBS, hM4Di, and hM3Dq). Moreover, the application of Student's t-test for multiple comparisons is inappropriate and warrants correction.

2. Regarding Supplementary Figure 1, it is essential for the authors to provide complete brain slice images to aid reviewers in accurately identifying the ACC. Additionally, concerns arise from the observation that the distribution of DREADD-mCherry expression appears to be non-uniform across ACC layers. Notably, layer 2/3 lacks hM3Dq expression, while deeper layers exhibit a deficiency in hM4Di expression. This inconsistency complicates the interpretation of behavioral outcomes, rendering all results inconclusive.

Round 2 Decision Letter
Thank you for your revised manuscript ID OXFNSC-2023-019.R1 entitled "Astrocytic GPCR signaling in the anterior cingulate cortex modulates decision making in rats". Your manuscript has been re-reviewed by the original reviewer 1. I have also read it myself and discussed it with the editorial team at Oxford Open Neuroscience. The comments of the reviewer are included at the bottom of this letter.

The reviewer has recommended publication, but also suggested some revisions to your manuscript.  Therefore, I invite you to respond to the reviewer comments and revise your manuscript within the next 60 days from todays date.

The reviewer has made two comments. I would like you to respond to the first of these, concerning Supplementary Figure 2.

Regarding the second comment, about Supplementary Figure 1, I am satisfied that the photomicrographs provided are sufficient to show good expression in ACC layer 2/3 and 5. Therefore, you should consider it optional whether to act on this and I am not making it a condition of acceptance.

Please note that this journal operates with transparent peer review. This means that if your submission is accepted for publication, the full peer review history of your article will publish online alongside your article. This includes reviewer comments, editor decision letters, and your author responses.

To revise your manuscript, log into https://mc.manuscriptcentral.com/oxfnsc and enter your Author Centre, where you will find your manuscript title listed under "Manuscripts with Decisions."  Under "Actions," click on "Create a Revision."  Your manuscript number has been appended to denote a revision.

You may also click the below link to start the revision process (or continue the process if you have already started your revision) for your manuscript. If you use the below link you will not be required to login to ScholarOne Manuscripts.

*** PLEASE NOTE: This is a two-step process. After clicking on the link, you will be directed to a webpage to confirm. ***

https://mc.manuscriptcentral.com/oxfnsc?URL_MASK=fdbf8dfe6ed644fdb8de5a37a48e166a

You will be unable to make your revisions on the originally submitted version of the manuscript.  Instead, revise your manuscript using a word processing program and save it on your computer.  Please also highlight the changes to your manuscript within the document by using the track changes mode in MS Word or by using bold or colored text.

Once the revised manuscript is prepared, you can upload it and submit it through your Author Centre.

When submitting your revised manuscript, you will be able to respond to the comments made by the reviewer(s) in the space provided.  You can use this space to document any changes you make to the original manuscript.  In order to expedite the processing of the revised manuscript, please be as specific as possible in your response to the reviewer(s).

IMPORTANT:  Your original files are available to you when you upload your revised manuscript.  Please delete any redundant files before completing the submission.

Because we are trying to facilitate timely publication of manuscripts submitted to the Oxford Open Neuroscience, your revised manuscript should be uploaded within the next 60 days from todays date.  If it is not possible for you to submit your revision in a reasonable amount of time, we may have to consider your paper as a new submission.

Once again, thank you for submitting your manuscript to the Oxford Open Neuroscience and I look forward to receiving your revision.

Final Decision Letter
It is a pleasure to accept your manuscript entitled "Astrocytic GPCR signaling in the anterior cingulate cortex modulates decision making in rats" in its current form for publication in the Oxford Open Neuroscience. 

Please note that this journal operates with transparent peer review. This means that the full peer review history of your article will publish online alongside your article. This includes reviewer comments, editor decision letters, and your author responses.

Next steps
You will receive an email from no-reply@scipris.com within roughly one week. This is your invitation to sign up for an account with SciPris, Oxford University Press’ author portal hosted by Aptara. You will need to create an account if you do not already hold one. Please register or log into your account and follow the online instructions which will guide you through signing your licence and paying the APC. The email and the portal have clearly signposted support options if you need any help during this process.

Please note that SciPris is a completely different system from ScholarOne, so your credentials to submit your manuscript here will not work there. Once you’ve created a SciPris account, you will be able to use it whenever you publish with Oxford Open Neuroscience or any OUP journal. Please note that OUP will only ever request payment for applicable fees be made via SciPris or to an OUP bank account. If you ever have concerns about the legitimacy of a request, please do not hesitate to contact a customer services agent via the SciPris portal or directly via oupsupport@scipris.com.

Thank you for your fine contribution.  On behalf of the Editors of the Oxford Open Neuroscience, we look forward to your continued contributions to the Journal.
